# Cell-specific expression of lung disease risk-related genes in the human small airway epithelium

**DOI:** 10.1186/s12931-020-01442-9

**Published:** 2020-07-29

**Authors:** Wu-lin Zuo, Mahboubeh R. Rostami, Shushila A. Shenoy, Michelle G. LeBlanc, Jacqueline Salit, Yael Strulovici-Barel, Sarah L. O’Beirne, Robert J. Kaner, Philip L. Leopold, Jason G. Mezey, Juergen Schymeinsky, Karsten Quast, Sudha Visvanathan, Jay S. Fine, Matthew J. Thomas, Ronald G. Crystal

**Affiliations:** 1 Department of Genetic Medicine, Weill Cornell Medical College, 1300 York Avenue, Box 164, New York, NY 10065 USA; 2 Present address: The Jackson Laboratory for Genomic Medicine, Farmington, CT USA; 3 Department of Medicine, Weill Cornell Medical College, New York, NY USA; 4 Department of Biological Statistics and Computational Biology, Cornell University, Ithaca, NY USA; 5 Boehringer Ingelheim Pharma GmbH & Co. KG, Biberach an der Riss, Germany; 6 Boehringer Ingelheim Pharmaceuticals, Ridgefield, CT USA

**Keywords:** Single-cell transcriptomes, Epithelial cells, Immune/inflammatory cells, Inherited and acquired pulmonary disorders, Cigarette smoking

## Abstract

**Background:**

The human small airway epithelium (SAE) plays a central role in the early events in the pathogenesis of most inherited and acquired lung disorders. Little is known about the molecular phenotypes of the specific cell populations comprising the SAE in humans, and the contribution of SAE specific cell populations to the risk for lung diseases.

**Methods:**

Drop-seq single-cell RNA-sequencing was used to characterize the transcriptome of single cells from human SAE of nonsmokers and smokers by bronchoscopic brushing.

**Results:**

Eleven distinct cell populations were identified, including major and rare epithelial cells, and immune/inflammatory cells. There was cell type-specific expression of genes relevant to the risk of the inherited pulmonary disorders, genes associated with risk of chronic obstructive pulmonary disease and idiopathic pulmonary fibrosis and (non-mutated) driver genes for lung cancers. Cigarette smoking significantly altered the cell type-specific transcriptomes and disease risk-related genes.

**Conclusions:**

This data provides new insights into the possible contribution of specific lung cells to the pathogenesis of lung disorders.

## Introduction

The small airway epithelium (SAE), a single layer of cells covering the branching airways from the 6th-23^rd^ generations, plays a central role in the early events in the pathogenesis of most lung disorders, including hereditary lung disorders, chronic obstructive pulmonary disease (COPD), idiopathic pulmonary fibrosis (IPF) and lung cancers [1–4]. The human SAE is comprised of 5 major cell types, including basal (BC), intermediate, ciliated, mucin-producing and club cells [5, 6]. The SAE also harbors small numbers of rare epithelial and inflammatory/immune cells [7–9]. Little is known about the molecular phenotypes of the specific cell populations comprising the SAE, and the contribution of specific SAE cell populations to the pathogenesis of human lung disorders.

Using single-cell RNA-sequencing, we defined the transcriptomes of eleven small airway cell populations recovered by brushing the 10^th^–12^th^ order bronchi of nonsmokers and smokers. The data demonstrate cell-specific differences in genes essential to the risk for lung-related hereditary monogenic disorders, COPD, IPF, and lung cancers. For many of these hereditary and acquired disorders, the analysis uncovered unexpected cell specificity in both major and rare small airway cell types modulated by cigarette smoking.

## Methods

### Study population and biologic samples

Subjects were recruited under a protocol approved by the Weill Cornell Medicine Institutional Review Board (IRB #1204012331). The SAE was brushed from 10^th^–12^th^ generation bronchi by fiberoptic bronchoscopy of 3 healthy nonsmokers and 3 asymptomatic smokers (Supplemental Table S1). Single viable cells were obtained through the trypsinization and flow cytometry sorting of the brushed SAE. Drop-seq single-cell RNA-sequencing was performed and a total of 11,702 single cells were characterized. See Supplemental Methods for details.

## Results

### Transcriptomic heterogeneity in the human SAE

**Fig. 1:**
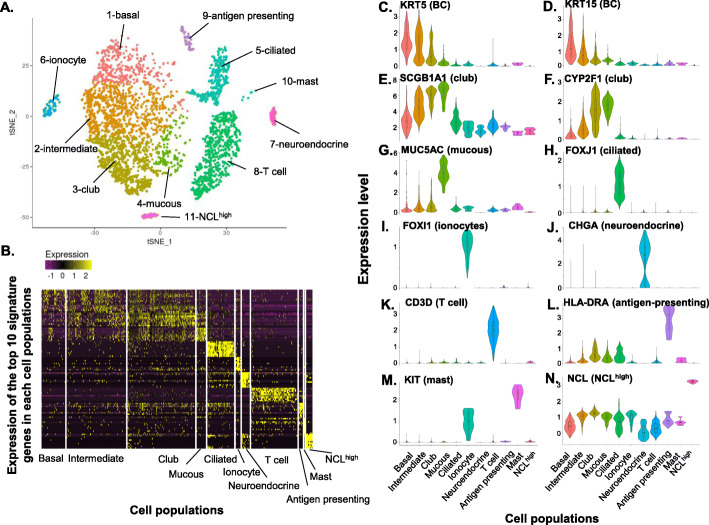
Single-cell RNA sequencing identification of 11 unique cell populations recovered by bronchoscopy and brushing of the nonsmoker human small airway epithelium (SAE). Drop-seq-based single-cell RNA sequencing was performed on cells recovered from 3 healthy nonsmokers; 4275 cells passed quality control filtering. **a** Unsupervised t-distributed Stochastic Neighbor Embedding (t-SNE) clustering. Based on signature genes (Supplemental Tables S2, S3, and S4) and known markers for these cell populations, these 11 cell populations were defined as: (1) basal cells; (2) intermediate cells; (3) club cells; (4) mucous cells; (5) ciliated cells; (6) ionocytes; (7) neuroendocrine cells; (8) T cells; (9) antigen presenting cells; (10) mast cells; and (11) unidentified NCL^high^ cells. The cell populations were identified in all 3 individuals, except: mast cells (population 10), observed in 2 of the 3 individuals; and neuroendocrine cells and the NCL^high^ cell population, which were identified in only one individual. **b** Heatmap plot displaying the expression of the top 10 signature genes from all 11 cell populations identified in panel **a.** The signature genes for each cell population are listed in Supplemental Tables S2, S3, and S4. **c-n** Violin plots of the markers for different cell types in the cells populations identified in panel **a** The violin plots were constructed with imputed data. **c.** KRT5 (*p* = 4.7 × 10^− 41^), basal cell; **d** KRT15 (*p* = 9.9 × 10^− 62^), basal cell; **e.** SCGB1A1 (*p* = 4.0 × 10^− 179^), club cells; **f** CYP2F1 (*p* = 8.6 × 10^− 183^), club cells; **g** MUC5AC (*p* = 1.1 × 10^− 146^), mucous cells; **h** FOXJ1 (*p* = 1.9 × 10^− 164^), ciliated cells; **i** FOXI1 (*p* = 3.5 × 10^− 52^), ionocytes; **j** CHGA (*p* = 6.3 × 10^− 142^), neuroendocrine cells; **k** CD3D (*p* = 1.5 × 10^− 291^), T cells; **l** HLA-DRA (*p* = 9.7 × 10^− 130^), MHC class II, antigen presenting cells; **m** KIT (*p* = 5.5 × 10^− 4^), mast cells; and **n** NCL (p = 3.5 × 10^− 161^). The *p* values, adjusted by Bonferroni correction, were generated by comparison of the indicated cell population vs all other cells in the total population (see Supplemental Table S2)

To assess cell-specific heterogeneity of gene expression in the human SAE, a total of 4275 single cells from 3 nonsmokers were sequenced and analyzed. The unsupervised t-SNE clustering of the single-cell transcriptomes identified 11 unique cell populations (Fig. 1a-b, Supplemental Figure S1), including: 1 – BC, highly expressing KRT5, KRT15 and TP63; 2 – intermediate cells, highly expressing both BC (KRT5, KRT15, TP63), and club cell (SCGB1A1, CYP2F1) markers; 3 – club cells, highly expressing SCGB1A1 and CYP2F1; 4 – mucin-producing cells, highly expressing MUC5AC; 5 – ciliated cells, highly expressing FOXJ1; 6 – ionocytes, highly expressing FOXI1; 7 – neuroendocrine, highly expressing CHGA; 8 – T cells, highly expressing CD3D; 9 – antigen-presenting cells, highly expressing major histocompatibility complexes (MHCs), including HLA-DRA, 10 – mast cells, highly expressing KIT; and 11 – NCL^high^ cells, highly expressing NCL, a gene encoding a nucleolar protein (Fig. 1c-n, Supplemental Figure S1, Supplemental Table S2). While some clusters had a distinct border in the tSNE plot (e.g., ionocytes and ciliated cells), other clusters merged with each other and lacked clear borders (Fig. 1a), likely indicating that the two clusters may share a differentiation route. Due to the various tolerances to enzyme digestion and cell processing, the fractions of different cell populations may not represent the original proportions of differentiated epithelial cells, but the transcriptomic information for the cell types should not be altered. An example of this phenomenon can be seen in the relative contribution of basal cells and ciliated cells to the overall population of epithelial cells. Immediately after brushing, cell differentials show that approximately 60% of cells are ciliated cells while basal cells represent approximately 2% of the population (see Supplemental Table S1). However, in the single cell dataset, the number of basal cells often exceeds the number of ciliated cells (see Supplemental Table S10) suggesting that small round basal cells endure the cell processing to a greater degree than large, elongated ciliated cells.

Transcriptomic analysis of the signature genes for the major epithelial cell populations demonstrated that BC highly expressed the genes related to cytoskeleton (KRT15, HSPB1, KRT5), barrier integrity (PERP, CLDN1), growth factors (IL33), and many ribosomal genes. Consistent with our previous study [10], club cells served as the “host defense” cells with abundant expression of genes in defense against pathogens and particulates (SCGB1A1, C3, LCN2), immunity-related receptors (PIGR), defense against toxins (MGST1, ALDH1A1) and anti-proteases (SLPI, WFDC2). The club cells also expressed high levels of protease-related genes (PRSS23, CTSC), that together with anti-proteases, are important for the susceptibility to viral infection [11]. The intermediate cells, localized between BC and club cells (Fig. 1a), expressed both basal and club cell genes. The mucin-producing cells had a similar transcriptome as club cells in host defense functions, but with additional expression of mucous-related genes (TFF3, MUC5AC). As expected, ciliated cells expressed genes relevant to ciliogenesis and ciliary architecture (Supplemental Tables S2 and S3).

The single-cell RNA-sequencing also uncovered novel insights into minor cell populations in the human SAE. There are ionocytes in the SAE, a rare cell population recently identified in the mouse airways and human large airway epithelium (LAE) [12, 13]. In the human SAE, the ionocytes functions related to ionic transport, phagosome acidification and insulin receptor signaling (Supplemental Figure S2A). Like ionocytes in mouse airways and human LAE [12, 13], the human SAE ionocytes highly expressed the transcription factors FOXI1 and ASCL3, V-ATPase-subunit genes (ATP6V1G3, ATP6V0B), and the Cl^−^ ion channel CFTR, that when mutated, causes cystic fibrosis (CF). However, in contrast to the ionocytes in the human LAE, the human SAE ionocytes had a unique expression of genes-related to other ion channels (GABRB2, SCN9A), defense against toxins (DGKI), cell surface receptors (KIT), extracellular matrix ligands (POSTN) and the cyclic nucleotide phosphodiesterase specific for cAMP and cGMP (PDE1C, PDE11A; Fig. 1m, Supplemental Figure E2B-C, Supplemental Tables S2 and S4).

The SAE also contained a small number of neuroendocrine cells, specialized epithelial cells known to be present throughout human airways [7, 14], with high expression of microtubules (TUBA4A, TUBA1A) and neuroendocrine mediators (RTN1, CHGA). The neuroendocrine cells also expressed calmodulin genes (CALM1–3) involved in Ca^2+^ signaling transduction [15], and GNG13, GNAL and RIC8B important for taste and odorant signaling [16–18] (Supplemental Tables S2 and S4). Neuroendocrine cells were only detected in one out of three nonsmoker samples. This result was not unexpected since neuroendocrine cells represent a small proportion of total airway cells (< 1%) and their distribution along the airway is not homogenous [19, 20].

In addition to the epithelial cell populations, the human SAE harbors inflammatory/immune cells, including T cells expressing a variety of cell surface molecules (CD2, CD3, MHC class I molecules) and cytokines (CCL5, IL32). Also present were mast cells, cells that play a central role in allergic responses [7–9]. Mast cell signature genes (SRGN, LAPTM5, TYROBP, KIT, CD52) play a role in protein secretion, signal transduction, and receptors. In addition, a variety of MHC class II molecules were highly expressed in the antigen-presenting cells, as well as some defense genes (LYZ, CYBB). The NCL^high^ cells were not well defined. This cell population highly expressed genes-associated with structural elements (ACTA2, TUBA4A) and the cell cycle (CDC5L, CDC37). Interestingly, the NCL^high^ cell population also highly expressed the pluripotent stem cell transcription factors (KLF4, SOX2) and genesrelated to histone modification (EZH2, HDAC1; Supplemental Tables S2 and S4). Lastly, the expression of MKI67, a proliferation marker, was very low in BC, while relatively higher in the intermediate, club, T and antigen-presenting cells (Supplemental Figure S1B).

#### Expression of the genes-associated with the risk for lung disorders

Little is known about the contribution of specific SAE cell populations to the pathogenesis of inherited and acquired lung disorders. As an initial approach to answer this question, we assessed the single-cell data for expression of genes known to be associated with a risk for monogenetic lung disorders, COPD, IPF and lung cancers.

##### Monogenetic lung disorder-related genes

**Fig. 2:**
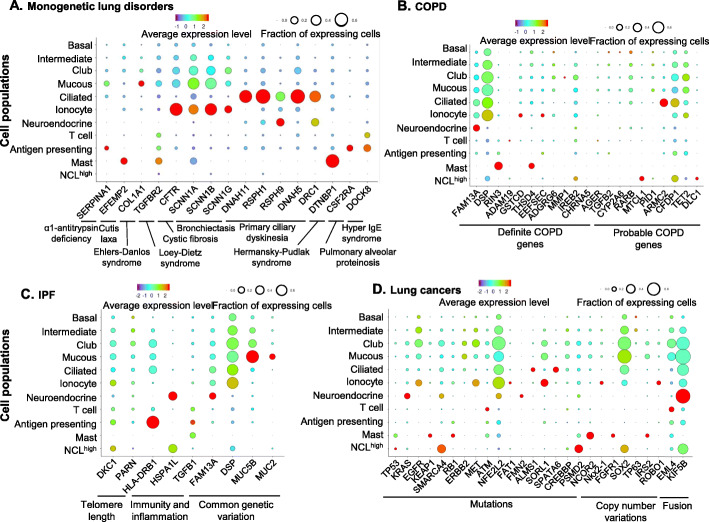
Dot plots of expression of specific small airway cell genes associated with lung disorders. **a** Monogenic lung disorder genes (see Supplemental Figure S3 for the full gene list). **b** Genes related to COPD identified by COPD genome-wide association studies (GWAS) or exome chip and GWAS of COPD-related phenotypes (http://www.copdgene.org). Shown are definite COPD risk genes (left) and probable COPD risk genes (right). The COPD-related Mendelian disorder COPD-related genes SERPINA1, ELN and FBLN5 are shown in Figure 2a and Supplemental Figure S3; **c.** IPF-related genes (see Supplemental Figure S5 for the full gene list). **d** Lung cancer-related genes (The Cancer Genome Atlas - TCGA). If mutated, increased copy number or fused with other genes, these genes can become cancer driver genes. For **a-d** The identities of the cell populations are shown on the y-axis, and the gene symbol and detailed categories of the genes are shown on the x-axis. The size of the dots represents the fraction of the expressing cells in each cell population. The color represents the average gene expression in positive cells

Single-cell RNA-sequencing of the SAE of healthy individuals demonstrated expression of genes that, when mutated, are responsible for monogenetic lung disorders (see Fig. 2a for examples; see Supplemental Figure S3 for details). We and others have previously shown that CFTR, the causative gene for CF, is expressed broadly in airway epithelial cells [10, 12, 13, 21]. Consistent with our previous data, here we observed that CFTR was widely distributed in the club cells, as well as intermediate and mucous cells, with intermediate fractions and expression levels. Our data also identified a small proportion of cells with high CFTR expression in a high percentage of cell, previously designated as ionocytes [12, 13]. In addition, SAE ionocytes also expressed the high levels of epithelial Na^+^ channel genes (SCNN1A, SCNN1B, SCNN1G; Fig. 2a, Supplemental Figure S3C, H-I), risk genes relevant to CF and bronchiectasis [4, 22]. As expected, expression of most primary ciliary dyskinesia (PCD)-related genes were highly enriched in the ciliated cells, with low expression in small fractions of the other cell populations. High expression of some of the PCD genes (RSPH9, DRC1) were also observed to some extent in the neuroendocrine cells (Fig. 2a, Supplemental Figure S3A-B, J). Some other monogenetic lung disorder-related genes were enriched in specific SAE cell types, including BC (LTBP4, ELN, cutis laxa), ionoctyes (HPS5, Hermansky-Pudlak syndrome), mucin-producing cells (COL1A1, Ehlers-Danlos syndrome), and neuroendocrine cells (BMPR2, pulmonary hypertension; Supplemental Figure S2D-F). Interestingly, some immune cells residing in the SAE also had high expression of monogenetic lung disorder genes. SERPINA1, that when mutated causes α1-antitrypsin deficiency, was expressed in the antigen-presenting cells. Cutis laxa (EFEMP2) and Hermansky-Pudlak syndrome (DTNBP1)-related genes were enriched in mast cells. DOCK8 (hyper IgE syndrome) was expressed in antigen presenting and T cells (Supplemental Figure S3D, G-K).

##### COPD-related genes

COPD risk genes, identified by genome-wide association studies (GWAS)/exome chip or related phenotypes, were grouped as definite (16 genes) and probable (10 genes) COPD risk-related genes (http://www.copdgene.org/). Twelve of the definite and 8 of the probable COPD-risk genes were detected in a variety of SAE cell populations (Fig. 2b, Supplemental Figure S4). Overall, most of the COPD genes were expressed in very small subsets of the SAE cell populations, except DSP, a gene that anchors intermediate filaments [23], which was expressed in ~ 60% of the major differentiated epithelial cells and ionocytes. FAM13A, a definite COPD gene functions in β-catenin degradation [24], was expressed in low levels of most epithelial cells, but in high level in the neuroendocrine cells. ARMC2, a probable COPD gene and also a PCD gene, was enriched in ciliated cells. TET2, a key gene in DNA demethylation, was primarily expressed in the intermediate and secretory cells. Interestingly, the probable COPD gene CFDP1 was expressed in the subset of NCL^high^ cells, as well as in the major epithelial cell populations (Fig 2a, Supplemental Figure S3 D-F, G, K, L).

##### IPF-related genes

**Fig. 3:**
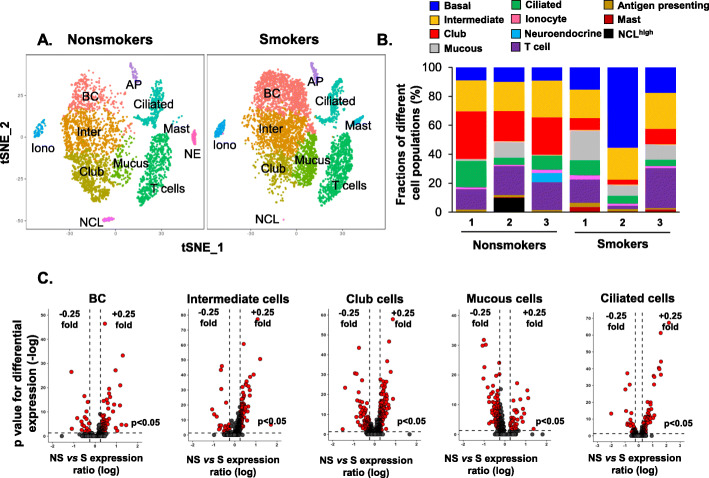
Impact of cigarette smoking on gene expression in specific cell populations in the human small airway epithelium. **a** Unsupervised t-SNE clustering identifies unique corresponding cell populations in nonsmokers (left) vs smokers (right). The names or abbreviations of different cell populations were labeled in the figure. Full names of the abbreviations as followed: BC – basal cells; Inter – intermediate cells; Iono – ionocytes; NE – neuroendocrine cells; AP – antigen-presenting cells; NCL – NCL^high^ cells. No neuroendocrine cells were identified in smokers. **b** Fractions of different cell populations in nonsmokers vs smokers in each individual, nonsmoker (*n* = 3) vs smoker (n = 3). **c** Volcano plots of significantly down-regulated (left) and up-regulated (right) genes in smokers (S) vs nonsmokers (NS) in SAE individual cell types, including basal cells, intermediate cells, club cells, mucous cells and ciliated cells. Y-axis represents the negative p value (log) and the x-axis represents the fold-change (log). The cutoff is shown as dotted lines. Fold-change (log) > 0.25 for the up-regulated genes or < − 0.25 for the down-regulated genes, *p* < 0.05 with Bonferroni correction

GWAS studies have identified that alveolar surfactant, telomere length, and inflammatory genes may be involved in the risk for IPF [25–27]. Assessment of the SAE single-cell data showed that DKC1 and PARN, genes-related to telomere length, were expressed in subsets of the major epithelial cells populations, and a few immune-cells (Fig. 3c, Supplemental Figure S5A). Several inflammatory genes related to IPF risk were also expressed in the SAE, including HSPA1L and TGFB1 in neuroendocrine and antigen-presenting cells, respectively (Fig. 3c, Supplemental Figure S5B, F, H). The MUC5B promoter variant rs35705950, is one of the strongest genetic risk factors related to IPF [28]. Interestingly, MUC5B was one of the top signature genes for the intermediate, club and mucin-producing cells (Fig. 3c, Supplemental Figure S5C-D). Other genes with polymorphisms associated with the risk for IPF were expressed in subsets of the various cell populations, including CDKN1A and HLA-DRB1 in antigen-presenting cells, and MUC2 in mucin-producing cells (Fig. 3c, Supplemental Figure S5C, E, G, I). The IPF genes DSP and FAM13A are also associated with increased risk for COPD (Fig. 2b-c, Supplemental Figures. S4A-B, 5C), suggesting a relationship between the two diseases.

##### Lung cancer-related genes

Mutations of most “driver” genes causative of lung cancers are in the coding sequence of the gene [29, 30]. Single-cell analysis of the SAE from nonsmokers demonstrated the cell-specific expression of the potential (if mutated) driver genes for lung cancers in human SAE (Fig. 2d, Supplemental Figure S6). EGFR, mutated in 10 ~ 35% non-small cell lung cancers (NSCLC) [31], was enriched in BC, intermediate cells, and at a higher level in ionocytes. KRAS, the oncogene causing 10 ~ 30% of lung adenocarcinomas [31], was highly expressed in the neuroendocrine cells. MET, mutated in NSCLC [31], was a signature gene for both intermediate and club cells. TP53, the most widely mutated gene in lung cancers [31–34], and RB1, a well-defined mutated gene in small cell lung cancer [33, 34], were expressed in small subsets of the NCL^high^ cells and mast cells, respectively (Fig. 2d, Supplemental Figure S6A-D).

In addition to single nucleotide mutations, copy number variation and oncogene fusion also contribute to the progression of lung cancers [31–35]. The fusion-related genes EML4 and KIF5B were both expressed in the major epithelial cells in human SAE. In addition, EML4 was also enriched in T cells, and KLF5B was enriched in the neuroendocrine cells (Fig. 2d, Supplemental Figure S6E). SOX2, a common gene amplified in lung cancer, was expressed in the epithelial cells and enriched in the NCL^high^ cells (Fig. 2d, Supplemental Figure S6F), suggesting a possible role of the undefined NCL^high^ cell population as the cell origin of some lung cancers.

### Cigarette smoking alters the Transcriptomes of specific SAE cell populations

The transcriptome of the human SAE is significantly dysregulated in smokers [36], but little is known regarding the impact of smoking on the transcriptomes of specific SAE cell populations*.* To answer this question, 6977 single cells from the human SAE of 3 smokers were sequenced, analyzed, and compared to the single cells from nonsmokers. Ten cell populations, except neuroendocrine cells, were identified in the smokers and mapped to the cell populations in nonsmokers (Fig. 3a). Compared to the nonsmokers, the fraction of club cells significantly decreased, while fractions of BC, mucin-producing and mast cells increased in smokers (Fig. 3b), consistent with smoking-relevant morphological changes in human airways [1, 5, 37].

**Fig. 4:**
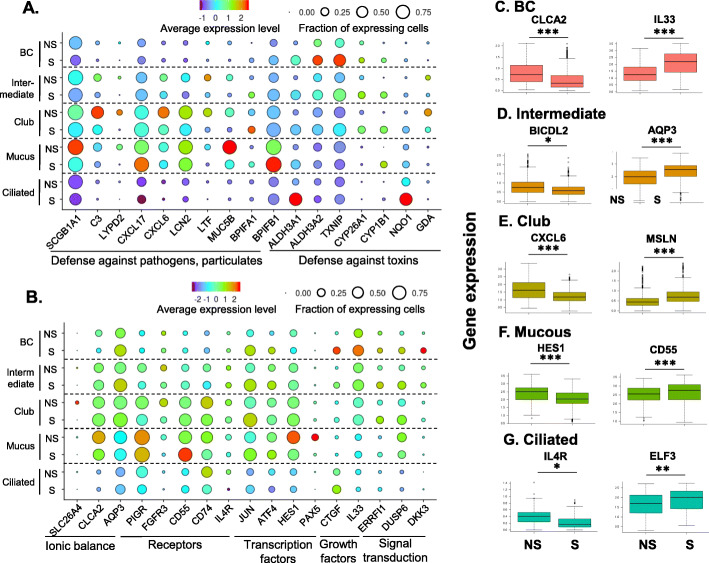
Impact of cigarette smoking on gene expression of specific cell populations in the human small airway epithelium. **a**, **b** Dot plots of functional categories of cigarette smoking-associated dysregulated genes. The identities of the cell populations are shown on the y-axis, and the functional categories are shown on the x-axis. NS – nonsmokers; S – smokers. The size of the dots represents the fraction of the expressing cells in each cell population, and the color intensity represents the average expression level. **c-j** Box plots of the cell type-specific down-regulated (left) or up-regulated genes (right) genes in smokers. The data are derived from the single-cell RNA-sequencing dataset. The cell types are labeled on the top of each panel. The box plots were constructed using imputed data. **c** basal cells, *left* – CLCA2, *right* – IL33; **d** Intermediate cells, *left* – BICDL2, *right* – AQP3; **e** Club cells, *left* – CXCL6, *right* – MSLN; **f** Mucous cells, *left* – HES1, *right* – CD55; and **g** Ciliated cells, *left* – IL4R, *right* – ELF3. * p < 0.05, ** *p* < 0.01 and *** *p* < 0.001 with Bonferroni correction

Comparing the transcriptomes of each cell population in nonsmokers vs smokers, smoking significantly altered the transcriptomes of the major epithelial cell populations (Fig. 3c), while the effects were less dramatic in ionocytes and immune cells (Supplemental Figure S7). Consistent with the changes at bulk SAE level, genes-related to defense against toxins (e.g., ALDH3A1, CYP1B1) and defense against pathogens and particulates (e.g., SCGB1A1, LTF) were up and down-regulated in the major epithelial cell populations in smokers, respectively (Fig. 4a, Supplemental Tables S5 and S6). Some novel defense genes were dysregulated in specific cell populations, e.g. CXCL6, chemoattractant for neutrophils, and ALCAM, member of the immunoglobulin superfamily, were down-regulated in club and ciliated cells of smokers, respectively, suggesting the loss of host defense in SAE of smokers are heterogeneous among different cell types. The mucin-related genes MUC5AC and AGR2 were up-regulated by smoking in the mucin-producing cells, but not in the club cells (Fig. 4a, Supplemental Tables S5 and S6).

Other effects of smoking on cell-specific gene expression included: (1) transcriptional regulation (ATF4 - increased in BC; PAX5, HES1 - decreased in mucin-producing cells); (2) growth factors (CTGF, IL33 - increased in BC); (3) receptors (CD55 - increased in mucous-producing cells; PIGR – decreased in intermediate, club and ciliated cells); (4) ionic balance (CLCA2 – decreased in BC; AQP3 – increased in intermediate cells); and (5) signal transduction (ERRFI1 - increased in intermediate and club cells; DKK3 - increased in BC; Fig. 4b, Supplemental Tables S5 and S6). Many important functional genes were dysregulated by smoking in only one specific cell population (see Fig. 4c-g for examples).

Duclos et al. [38] described two ciliated cell sub-populations in human LAE with one sub-population characterized by expression of cilia-related genes while the other sub-population was characterized by expression of cell cycle genes and transcription factors. To better understand the cigarette smoking-induced transcriptional heterogeneity in the ciliated cells of the human SAE, we re-analyzed the ciliated cell population (Fig. 1a, cluster 5) and a total of 4 ciliated cell sub-populations were identified (Supplemental Figure S8A). The fractions of ciliated cell sub-population 2 and 4 were not changed in nonsmokers vs smokers, and the ciliated cell sub-population 1 was mostly contributed by one nonsmoker. Interestingly, the fraction of ciliated cell sub-population 3 was significantly increased in the smokers (Supplemental Figure S8B, Supplemental Table S7). Transcriptomic analysis showed that the common ciliated cell-related genes (FOXJ1, DNAH9, CDHR3, IFT88 and DNAH5) were evenly expressed in all the ciliated cell subpopulations, while the aldehyde and ketone metabolism-related genes (ALDH3A1, AKR1C1, ADH7, NQO1, AKR1B10, PRDX1, AKR1C3 and ALDH1A1) were highly expressed in the ciliated cell sub-population 3. The cell cycle-related genes (CDK1, CCNB1 and TOP2A) and transcription factor HES6 were only expressed in very small fractions of all 4 ciliated cell sub-populations (Supplemental Figure S8C).

Despite the inclusion of cells from only 3 donors for each condition, nonsmokers and smokers, the dataset appears to be robust. Individual tSNE plots for each sample confirmed that each sample was composed of similar overall clusters with substantial overlap among samples; each of the individual samples included ionocytes showing that even minor cells populations were included and overlapping in each sample (Supplemental Figure S9). In addition, the observed changes in gene expression permit validation within the dataset. For example, HES1 encodes a transcription factor that acts as a negative regulator of the Notch signaling pathway. In airway mucus cells, HES1 expression inhibits expression of MUC5AC [39]. HES1 was downregulated in mucus cells in smokers compared with nonsmokers in this dataset. Therefore, it stands to reason that mucus cells should also exhibit an increase in MUC5AC. In fact, MUC5AC was highly upregulated in the mucus cells of smokers (Fig. 4a; Supplemental Tables S5 and S6), providing support for the validity of the dataset.

### Effect of cigarette smoking on the cell-specific expression of lung disease risk genes

**Fig. 5:**
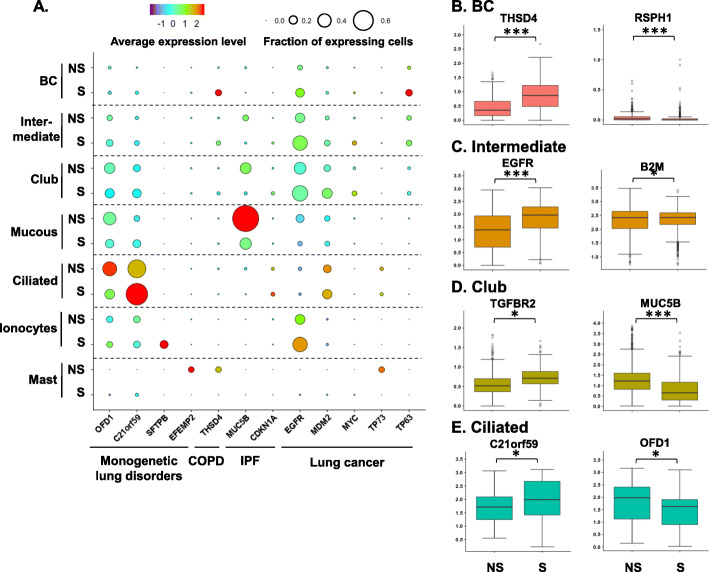
Smoking-induced changes in the expression of specific human small airway genes related to lung diseases. **a** Dot plot of expression of lung disease-related genes in the cell populations recovered by brushing small airway epithelium from healthy nonsmokers vs smokers. The categories of the lung diseases are shown on the x-axis and the identities of the cell populations on the y-axis. The size of the dots represents the fraction of the expressing cells in each cell population, and the color intensity represents the average expression level. **b-f** Box plots of the examples of lung disease-related genes in the major human SAE cell populations. The data are derived from the single-cell RNA-sequencing dataset. The cell types are labeled on the top of each panel. The box plots were constructed using imputed data. **b** basal cells; **c** intermediate cells; **d** club cells; and **e** ciliated cells. Gene symbols are shown in the Fig. NS – nonsmokers, S – smokers. * p < 0.05, ** p < 0.01, *** p < 0.001 with Bonferroni correction

Cigarette smoking is the leading cause of COPD and lung cancers, is a significant risk factor for IPF [1–3, 40], and worsens the severity of some inherited lung disorders [4]. In our analysis, definite COPD gene THSD4 was specifically up-regulated in BC, while, the IPF-related gene MUC5B was down-regulated in the intermediate, club and mucous-producing cells in smokers. Many lung cancers (when mutated)-related genes were up-regulated by smoking in specific cell populations, including TP63 in BC and EGFR in intermediate cells (Fig. 5a-d, Supplemental Table S8).

Several genes associated with monogenetic lung disorders were also dysregulated in specific cell populations of smokers, including PCD (OFD1 – down-regulated in ciliated cells) and surfactant deficiency (SFTPB - up-regulated in ionocytes)-related genes (Fig. 5a, e, Supplemental Table S8). Strikingly, expression of some of the lung cancer-related genes were dysregulated in immune cells of smokers, such as TP73 which was decreased in mast cells (Fig. 5a).

## Discussion

The complex molecular phenotypes and functions of the specific human SAE cells and their contributions to the genetic risk for lung disorders are not well defined. Using single-cell RNA-sequencing data, we characterized the transcriptomes of the epithelial and immune/inflammatory cells in the human SAE. Importantly, we were able to identify cell type-specific expression of genes associated with the risk for hereditary and acquired lung disorders. Many of these genes are modulated by smoking, the major risk factor for many lung diseases.

### SAE cell populations in nonsmokers

The single-cell transcriptome analysis identified eleven distinct cell populations in the SAE of nonsmokers, including 5 major epithelial cell populations (BC, intermediate, club, mucin-producing and ciliated cells) and 6 less common cell populations (ionocytes, neuroendocrine, T cells, antigen-presenting, mast, and undefined NCL^high^ cells). Consistent with our previous study [10], BC highly expressed ribosome and cytoskeleton-related genes, while club cells highly expressed the host defense-related genes. As expected, ciliated and mucin-producing cells expressed genes relevant to cilia architecture and mucus production, respectively. This study, using Drop-seq technology, includes some subtle differences from our prior single cell sequencing study that employed Fluidigm technology [10]. In the present study, a greater number of cells was sampled, but at a lower number of reads per cell. These technologies, as well as other single cell RNA sequencing methods (e.g., 10x Chromium), necessarily provide overlapping and complementary datasets that will vary as a function of technology and experimental methods. However, the remarkable agreement of the major features of the data, combined with the ubiquitous need for validation through other techniques, serves to solidify the importance of single cell RNA seq as an informative technology.

Ionocytes, originally identified in the skin of *Xenopus* and zebrafish [41, 42], have been described in the murine airway epithelium and human LAE [12, 13]. In our data, ionocytes are present in human SAE. Although rare, the SAE ionocytes highly express CFTR, the gene causative of cystic fibrosis if mutated [43]. In addition, SAE ionocytes express high level of other Cl^−^ channel and epithelial Na^+^ channel genes, genes also involved in the pathogenesis of CF [22] and bronchiectasis [4]. Interestingly, the SAE ionocytes highly express genes for the hydrolysis of cAMP, a second messenger to regulate CFTR channel gating [43]. Compared to the ionocytes in human LAE [12], SAE ionocytes have unique signatures, such as POSTN, a ligand for integrins [44]. POSTN functions as the downstream of IL13 and a biomarker for Th2-driven asthma [45], suggesting that SAE ionocytes may also be related to the pathogenesis of asthma.

Neuroendocrine cells represent a rare epithelial cell population localized throughout the entire conducting airways [7, 14]. In the human SAE, neuroendocrine cells uniquely expressed genes associated with neuroendocrine secretion, cytoskeleton and energy homeostasis. Also, they express high levels of taste and odorant signaling transduction-related genes and may serve as human SAE sensory cells. The neuroendocrine cells in this analysis were identified from only a single nonsmoker subject (Supplemental Table S10), likely a consequence of non-homogenous distribution in the airway [20]. Murine airways contain “tuft cells,” notable for their enrichment in taste receptors [12]. We did not detect “tuft cells” in human SAE. The lack detection of tuft cells does not mean they do not exist in humans. Tuft cells may be particularly fragile and/or discretely localized, which could account for their lack of detection from this study.

The single-cell analysis also identified several other notable cell clusters. Immune cells were prominently featured in the SAE, likely due to ove–representation as a result of high survival in the cell isolation procedure due to their native size, shape, and single cell status. These cells, together with the epithelial cells, create a unique niche, that likely contributes to homeostasis in human SAE [7–9]. Proliferating cells made up another group of cells in the single cell analysis. The relatively high expression of MKI67 expression in intermediate, club, T and antigen-presenting cells suggests a proliferative sub-population may exist in those cell populations. Finally, a novel population of cells marked by high expression of NCL was identified. The exact function of NCL^high^ cells remains elusive.

### Expression of lung disease genetic risk genes by specific SAE cell populations

The single-cell data also demonstrates that specific SAE cells likely play a role in the pathogenesis of hereditary human lung disorders if they harbor certain mutated genes. Notably, SERPINA1, causal gene for α1-antitrypsin deficiency if mutated [46], is expressed in epithelial (club, mucin-producing and ciliated cells) and antigen-presenting cells, suggesting that multiple cell types may contribute to the pathogenesis of α1-antitrypsin deficiency.

In addition to the likely role of ionocytes in CF [12, 13], rare small airway epithelial cells may also participate in the pathogenesis of other lung disorders by expression of disease associated genes, including monogenetic lung disorder-related genes in ionocytes, and COPD and IPF-related genes in the neuroendocrine cells. Interestingly, many lung cancer “driver” genes were highly expressed in the rare epithelial cells, with expression of EGFR and KRAS, the top mutated oncogenes in adenocarcinoma [31], enriched in ionocytes and neuroendocrine cells, respectively. The expression of the oncogenes in the rare epithelial cells suggests possible novel cell origins of lung cancers.

### Effects of cigarette smoking on gene expression of SAE specific cell types

Overall, the SAE transcriptome is significantly dysregulated by smoking [1, 2, 5, 36], but there is little information of the in vivo effects of smoking on specific cell populations. Morphologic strategies have identified the effects of smoking on some specific epithelial cell populations, such as a decrease of SCGB1A1^+^ club cells in smokers [5]. RNA-sequencing of BC, purified from the human SAE, identified a marked dysregulation of the BC transcriptome in smokers [47]. The single-cell RNA-sequencing of the SAE revealed that smoking significantly alters the molecular phenotypes and functions of specific epithelial cell populations. For example, expression of IL33, important for Th2 cytokine expression, is enriched in BC, and the numbers of IL33^+^ BC increases in COPD [48]. The single-cell analysis identified that the expression level of IL33 was also up-regulated in the BC of smokers, providing a specific target for drug development. Another interesting observation is the smoking effects on ionocytes, with down-regulation of Ca^2+^-sensitive Cl^−^ channel BEST3 and up-regulation of the of surfactant protein B, suggesting the functional roles of smoking on the ionic transport and surface tension in ionocytes.

Two ciliated cell subpopulations have been identified in the human LAE of never and current smokers [38]. Both ciliated cell subpopulations expressed common cilia-related genes, but had unique differential gene expression patterns. One subpopulation was more related to ciliary biology, while the other subpopulation was enriched with cell cycle-associated genes. Moreover, cigarette smoking induced a “detoxification” program in one of ciliated cell subpopulations, with genes associated with aldehyde and ketone metabolism up-regulated. Consistent with the finding in the LAE, the “detoxification” program was also enhanced in one ciliated cell sub-population of SAE in the smokers, suggesting both large and small airways share a similar ciliated cell subpopulation-dependent mechanism to protect the airways against the toxins from cigarette smoking. The lack of cell cycle-related ciliated cell sub-population in human SAE may reflect the differences of human large and small airways.

The dataset analyzed in this study is derived from three nonsmokers and three smokers. A larger number of donors would provide higher statistical power and would likely reveal additional differentially expressed genes. The size does not invalidate the study, but should be interpreted as showing the differences between groups with the largest magnitude and fidelity. By virtue of random accrual of subjects, the dataset presented here contains only female nonsmokers and only male smokers. Nevertheless, we conclude that the dataset primarily captures changes due to smoking status rather than sex. From our prior studies of gene expression in smokers, the effect *of* size due to smoking is approximately 50- to 100-fold larger than the effect due to sex (see Supplemental Table S11 and associated references). Adding a larger number of study subjects and achieving a balance in sex in the two study subject groups would likely lead to minor changes in the list of genes that are significantly different between the two groups, but would not change the major observations or conclusions. While remaining cognizant of these limitations, the overall findings are consistent with prior publications, increase the resolution with which we observe gene expression changes at the cellular level in a complex tissue, and lend to internal validation based on known signaling pathways and consequences of changes in gene expression.

## Supplementary information

**Additional file 1.**

**Additional file 2.**

**Additional file 3.**

## Data Availability

The data have been submitted to the National Center for Biotechnology Information Gene Expression Omnibus. The following link has been created to allow review of the GSE123405 dataset: https://www.ncbi.nlm.nih.gov/geo/query/acc.cgi?acc=GSE123405.

## Abbreviations

BC: Basal cells
CF: Cystic fibrosis
COPD: Chronic obstructive pulmonary disease
GWAS: Genome-wide association studies
IPF: Idiopathic pulmonary fibrosis
IRB: Institutional Review Board
LAE: Large airway epithelium
MHC: Major histocompatibility complexes
NSCLC: Non-small cell lung cancers
PCD: Primary ciliary dyskinesia
SAE: Small airway epithelium
