# Supplementary material for: Cell-specific expression of lung disease risk-related genes in the human small airway epithelium
Source: Respir Res. 2020 Jul 29;21:200. doi: 10.1186/s12931-020-01442-9 (PMC7389881; doi:10.1186/s12931-020-01442-9)
Supplement: Supplementary file 1 — Additional file 1. [file 12931_2020_1442_MOESM1_ESM.pdf]

# Supplemental Methods

## Sampling and Single Cell Isolation

The small airway epithelium (SAE) was brushed from $10^{th}$-$12^{th}$ generation bronchi by fiberoptic bronchoscopy of 3 healthy nonsmokers and 3 asymptomatic smokers (Supplemental Table S1) using a protocol approved by Weill Cornell Medical College Institutional Review Board. The pooled cells were prepared for single cell sequencing based on standard methods [1]. The pooled cells were washed with Small Airway Growth Basal Medium (Lonza, Walkersville, MD) and incubated with ammonium chloride potassium lysing buffer (Gibco, Grand Island, NY) for 3 min to remove the red blood cells. The cells were then digested by trypsin/ethylenediaminetetraacetic acid (EDTA, 0.05%; Gibco) for 5 min, and neutralized by N'-2-hydroxyethylpiperazine-N'-2 ethanesulfonic acid (HEPES; Lonza) with 15% fetal bovine serum (Gibco). The cells were washed again and resuspended in 1 ml phosphate buffered saline, pH 7.4 (PBS; Gibco) with 0.01% bovine serum albumin (BSA; Jackson ImmunoResearch, West Grove, PA). Trypan blue (Gibco)-treated cell suspensions were assessed using a hemocytometer and the numbers of viable cells quantified. In all cases, >80% of the cells were single cells, with 70 to 80% cell viability. Cell suspensions were filtered through a 35 µm nylon mesh cell strainer snap cap (Falcon/Becton Dickinson Labware, Franklin Lakes, NJ), stained with 4',6-diamidino-2-phenylindole, dihydrochloride (DAPI, 1 µg/ml; Molecular Probes, Basel, Switzerland), and loaded on an Influx™ cell sorter (BD Biosciences, San Jose, CA; Flow Cytometry Core, Weill Cornell Medicine Flow Cytometry Core Facility). The single viable cells were sorted, resuspended in PBS/0.01% BSA and the sorted single cell suspension counted using Countess™ Automated Cell Counter (Invitrogen, Carlsbad, CA) adjusted to the final concentration of 100 to 120 cells/µl.

**Single Cell RNA-sequencing and Analysis**

Drop-seq-based single cell RNA-sequencing was performed in the Weill Cornell Genomics Core Facility following the protocol from McCarroll [2, 3]. The single cells and barcoded beads were encapsulated into oil-based droplets using a co-flow microfluidics device (FlowJEM, Toronto, Canada). Single cells in the droplets were lysed immediately and the mRNA released from the cell hybridized to the barcoded primers on the surface of the beads. The droplets were then harvested and broken with perfluorooctanol. cDNA synthesis and library preparation were performed [2], and cDNA libraries were sequenced on Illumina HiSeq 2500 instrument (Illumina, San Diego, CA). The 6 samples (nonsmoker 1-3, smoker 1-3) were processed at 6 different days, the library preparation and sequencing were performed on 4 technical batches: batch 1 – nonsmoker 1 + smoker 1; batch 2 – nonsmoker 2; batch 3 – nonsmoker 3 + smoker 2; batch 4 – smoker 3. The single cell data are available in Gene Expression Omnibus (GEO) site with accession number: GSE123405.

Clustering was performed using Seurat, an R package for single cell analysis[14]. Raw digital expression matrices containing transcript counts for each gene in each cell were generated separately for each sequencing experiment[15]. Raw data was filtered as: (1) genes expressed in no less than 10 cells and cells with no less than 200 genes detected were kept for subsequent analysis; and (2) cells were filtered out that had unique gene counts over $10^4$ or <200 and the % mitochondrial genes for each cell >0.25. The quality control for the single-cell RNA-sequencing data in each individual after filtering was shown in Supplemental Table S9.

A total of 11,702 cells from the 6 samples were combined and normalized by the total number of unique molecular identifiers (UMI) per cell, multiplied by a scaling factor ($10^4$) and then log transformed. Cell-cell variation was regressed out in gene expression driven by the number of detected molecules per cell as well as percent mitochondrial gene content. The mean

expression and dispersion (variance/mean) were calculated for each of the 19,748 genes detected across the entire dataset to identify the most variable genes. Genes were placed into bins based on average expression and a z score calculated for dispersion within each bin to identify outlier genes whose expression values were highly variable compared to genes with similar average expression. A lower cutoff of 0.1 for average gene expression and 1 for dispersion was used to identify 1,952 highly variable genes. Principal component analysis (PCA) was used to assess selected genes from 11,702 cells. The identified highly variable genes were used as input to the PCA to ensure robust identification of the primary structure of the data [4]. Twenty statistically significant principal components (PCs) were calculated. Two approaches were used to determine significant PCs for downstream analysis. First, standard deviations of the PCs were plotted to identify the cutoff where a clear elbow existed in the plot. Second, the PCs score of cells and genes were visualized to explore correlated gene sets. The PCs that had lowest sum weight scores for mitochondrial and ribosomal genes were selected. The PCs were used to project cells onto a two dimensional map using t-Distributed Stochastic Neighbor Embedding (t-SNE) [5] with perplexity parameter set to 30, allowing cells with similar expression signature genes and similar PC loading to localize near each other. To identify distinct cell clusters, a K-nearest neighbor (KNN), graph based on the Euclidean distance in PCA space, was constructed to iteratively group cells together. The resolution parameter in FindClusters function in Seurat package was set to 0.5. With this approach, 11 distinct clusters were identified and the cell numbers of each cell population from each individual were shown in Supplemental Table S10. These clusters were compared using a Wilcoxon rank-sum test to identify up-regulated signature genes in each cluster. The criteria used to define signature genes for each cluster included: (1) genes detected at a minimum of 10% of cells in the cluster and (2) genes with mean expression increased by 0.25

(log scale) in the cluster compared to all other clusters [6]. Ambient RNA contamination was estimated using SoupX (https://omictools.com/soupx-tool ). The fraction of ambient RNA contamination in nonsmokers was 6.2% while smoker data had 5.4% ambient RNA contamination. We also did the t-SNE plots on each sample (Supplemental Figure S9A), as well as on the 4 technical batches (Supplemental Figure S9B). Most of the cell populations in either different individual or the technical batches overlapped quite well, suggesting our samplings and experimental processing were consistent and no big variations between different individuals or batches.

To evaluate the effects of gender to the gene expression of human SAE, we -analyzed our previous microarray and RNA-seq datasets of the bulk human SAE. We found that gender had little effect, while smoking dominates changes in gene expression of human SAE (Supplemental Table S11).

**Imputation Method for Gene-Gene Interactions**

The Markov Affinity-based Graph Imputation of Cells (MAGIC) computational approach was used for recovering missing values of mRNA capture [7]. MAGIC starts with count matrix representing observed transcript counts of genes and cells. Using a graph-based method, a distance matrix was calculated in the Seurat package (SNN matrix). A Gaussian kernel function was applied to convert the distance matrix to an affinity matrix as a weighted adjacency matrix. A Markov transition matrix was created by row normalizing the affinity matrix, and the imputed expression values were calculated by multiplying the Markov transition matrix powered to diffusion time (t) by the distance matrix. Genes associated with monogenetic lung disorders [8], idiopathic pulmonary fibrosis (IPF) [9-11] and lung cancers [12-16] were collected from the literature, and chronic obstructive pulmonary disease (COPD)-related genes taken from the COPDGene study (http://www.copdgene.org/). Imputed data is presented in violin and box plots throughout the text. Imputed data was not used for tSNE plots, heatmaps, dot plots or any other

data representations.

**Statistical Analysis**

To identify signature genes in each of the 11 clusters, gene expression from each cluster was compared to the gene expression from all other cells of remaining clusters by using the Seurat "FindAllMarkers" function. The test used to identify markers was the two-sided, combined likelihood-ratio test with three degrees of freedom, designed for the sampling distributions of single cell gene expression as described in McDavid et al [6]. The criteria to define marker genes included: (1) marker genes differed by at least 0.25 (log-scale) between the mean expression in each group of cells; and (2) that genes were only tested if they were detected in a minimum fraction of 0.1 cells in either of the cell groups. Bonferroni correction was used to adjust p values by multiplying by the total gene number of the dataset.

## Supplemental References


1. Zuo WL, Shenoy SA, Li S, O'Beirne SL, Strulovici-Barel Y, Leopold PL, Wang G, Staudt MR, Walters MS, Mason C, et al: **Ontogeny and Biology of Human Small Airway Epithelial Club Cells.** *Am J Respir Crit Care Med* 2018.
2. Macosko EZ, Basu A, Satija R, Nemesh J, Shekhar K, Goldman M, Tirosh I, Bialas AR, Kamitaki N, Martersteck EM, et al: **Highly Parallel Genome-wide Expression Profiling of Individual Cells Using Nanoliter Droplets.** *Cell* 2015, **161:**1202-1214.
3. **Drop-seq Core Computational Protocol version 1.0.1 (6/11/15) Steve McCarroll's Lab, Harvard Medical School**
4. Shalek AK, Satija R, Adiconis X, Gertner RS, Gaublomme JT, Raychowdhury R, Schwartz S, Yosef N, Malboeuf C, Lu D, et al: **Single-cell transcriptomics reveals bimodality in expression and splicing in immune cells.** *Nature* 2013, **498:**236-240.
5. van der Maaten L, Hinton G: **Visualizing data using t-SNE.** *J Mach Learn Res* 2008, **9:**2579-2605.
6. McDavid A, Finak G, Chattopadyay PK, Dominguez M, Lamoreaux L, Ma SS, Roederer M, Gottardo R: **Data exploration, quality control and testing in single-cell qPCR-based gene expression experiments.** *Bioinformatics* 2013, **29:**461-467.
7. van Dijk D, Sharma R, Nainys J, Yim K, Kathail P, Carr AJ, Burdziak C, Moon KR, Chaffer CL, Pattabiraman D, et al: **Recovering Gene Interactions from Single-Cell Data Using Data Diffusion.** *Cell* 2018, **174:**716-729 e727.
8. Tilley AE, Staudt MR, Salit J, Van de Graaf B, Strulovici-Barel Y, Kaner RJ, Vincent T, Agosto-Perez F, Mezey JG, Raby BA, Crystal RG: **Cigarette Smoking Induces Changes in Airway Epithelial Expression of Genes Associated with Monogenic Lung Disorders.** *Am J Respir Crit Care Med* 2016, **193:**215-217.
9. Kaur A, Mathai SK, Schwartz DA: **Genetics in Idiopathic Pulmonary Fibrosis Pathogenesis, Prognosis, and Treatment.** *Front Med (Lausanne)* 2017, **4:**154.
10. Kropski JA, Blackwell TS, Loyd JE: **The genetic basis of idiopathic pulmonary fibrosis.** *Eur Respir J* 2015, **45:**1717-1727.
11. Zhou W, Wang Y: **Candidate genes of idiopathic pulmonary fibrosis: current evidence and research.** *Appl Clin Genet* 2016, **9:**5-13.
12. Campbell JD, Alexandrov A, Kim J, Wala J, Berger AH, Pedamallu CS, Shukla SA, Guo G, Brooks AN, Murray BA, et al: **Distinct patterns of somatic genome alterations in lung adenocarcinomas and squamous cell carcinomas.** *Nat Genet* 2016, **48:**607-616.
13. Cancer Genome Atlas Research N: **Comprehensive genomic characterization of squamous cell lung cancers.** *Nature* 2012, **489:**519-525.
14. George J, Lim JS, Jang SJ, Cun Y, Ozretic L, Kong G, Leenders F, Lu X, Fernandez-Cuesta L, Bosco G, et al: **Comprehensive genomic profiles of small cell lung cancer.** *Nature* 2015, **524:**47-53.
15. Kohno T, Nakaoku T, Tsuta K, Tsuchihara K, Matsumoto S, Yoh K, Goto K: **Beyond ALK-RET, ROS1 and other oncogene fusions in lung cancer.** *Transl Lung Cancer Res* 2015, **4:**156-164.
16. Peifer M, Fernandez-Cuesta L, Sos ML, George J, Seidel D, Kasper LH, Plenker D, Leenders F, Sun R, Zander T, et al: **Integrative genome analyses identify key somatic driver mutations of small-cell lung cancer.** *Nat Genet* 2012, **44:**1104-1110.
17. Jones PW, Quirk FH, Baveystock CM, Littlejohns P: **A self-complete measure of health status for chronic airflow limitation. The St. George's Respiratory Questionnaire.** *Am Rev Respir Dis* 1992, **145:**1321-1327.

18. Montoro DT, Haber AL, Biton M, Vinarsky V, Lin B, Birket SE, Yuan F, Chen S, Leung HM, Villoria J, et al: **A revised airway epithelial hierarchy includes CFTR-expressing ionocytes.** *Nature* 2018, **560:**319-324.

## Supplemental Table S1. Demographics of Nonsmokers and Smokers[1, 2]

| Parameter | Nonsmokers | Smokers | p value |
|---|---|---|---|
| n | 3 | 3 | |
| Gender (M/F) | 0/3 | 3/0 | p>0.1 |
| Age (yr) | 26 ± 8 | 42 ± 14 | p>0.1 |
| Race (B/W/H/O)[2] | 1/1/0/1 | 1/0/1/1 | p>0.8 |
| Body Mass Index | 23 ± 4 | 23 ± 4 | p>0.8 |
| Smoking history | | | |
| Age of initiation | NA | 20 ± 1 | NA |
| Pack years | NA | 12 ± 6 | NA |
| Urine nicotine (ng/ml)[3] | 0 | 844 ± 616 | NA |
| Urine cotinine (ng/ml)[3] | 0 | 936 ± 458 | NA |
| Carboxyhemoglobin (%) | 1.6 ± 0.1 | 2.3 ± 0.2 | p>0.1 |
| Pulmonary function parameters[4] | | | |
| FVC | 115 ± 15 | 102 ± 13 | p>0.5 |
| FEV1 | 110 ± 12 | 100 ± 10 | p>0.4 |
| FEV1/FVC | 83 ± 5 | 80 ± 4 | p>0.4 |
| DLCO | 89 ± 8 | 92 ± 10 | p>0.1 |
| TLC | 107 ± 12 | 103 ± 6 | p>0.7 |
| Cough Score[5] | 0.7 ± 0.6 | 1.3 ± 0.6 | p>0.3 |
| Sputum Score[5] | 0.7 ± 0.6 | 1.3 ± 0.6 | p>0.3 |
| SAE cell differential (%) | | | |
| Epithelial | 96.6 ± 1.1 | 98.9 ± 0.2 | p>0.06 |
| Inflammatory | 3.4 ± 1.1 | 1.1 ± 0.2 | p>0.06 |
| Ciliated | 59.9 ± 2.0 | 57.7 ± 9.9 | p>0.7 |
| Secretory | 10.3 ± 2.5 | 9.6 ± 5.1 | p>0.8 |
| Undifferentiated | 24.0 ± 0.7 | 29.8 ± 5.8 | p>0.2 |
| Basal | 2.4 ± 1.0 | 1.8 ± 1.0 | p>0.4 |

[1] Data are presented as mean ± standard deviation, p values of numeric parameters calculated using a 2-tailed Student's t-test, p value of categorical parameters calculated using a Fisher's exact test.

[2] Abbreviations: B=Black, W=White, H=Hispanic, O=Other, NA=not applicable; FVC - forced vital capacity, FEV1 - forced expiratory volume in 1 sec, TLC - total lung capacity, DLCO - diffusing capacity of the lung for carbon monoxide, SAE=small airway epithelium.

[3] Undetectable urine nicotine <2 ng/ml; cotinine < 5 ng/ml.

[4] Pulmonary function testing parameters are given as % of predicted value with the exception of FEV1/FVC which is reported as % observed

[5] Cough and sputum score were each evaluated on a scale of 0-4: 0 = not at all; 1 = only with chest infections; 2 = a few days a month; 3 = several days a week; 4 - most days a week [17]

# Supplemental Table S2. Entire List of Signature Genes for the Cell Populations Identified by Unsupervised Clustering in Human Small Airways of Healthy Nonsmokers

| Basal | | | Intermediate | | | Club | | | Mucous | | | Ciliated | | | Ionocyte | | | Neuroendocrine | | | T cell | | | Antigen presenting | | | Mast | | | NCL$^{high}$ | | |
|---|---|---|---|---|---|---|---|---|---|---|---|---|---|---|---|---|---|---|---|---|---|---|---|---|---|---|---|---|---|---|---|---|
| Gene symbol | Fold-change ($log_e$) | Adjusted p value | Gene symbol | Fold-change ($log_e$) | Adjusted p value | Gene symbol | Fold-change ($log_e$) | Adjusted p value | Gene symbol | Fold-change ($log_e$) | Adjusted p value | Gene symbol | Fold-change ($log_e$) | Adjusted p value | Gene symbol | Fold-change ($log_e$) | Adjusted p value | Gene symbol | Fold-change ($log_e$) | Adjusted p value | Gene symbol | Fold-change ($log_e$) | Adjusted p value | Gene symbol | Fold-change ($log_e$) | Adjusted p value | Gene symbol | Fold-change ($log_e$) | Adjusted p value | Gene symbol | Fold-change ($log_e$) | Adjusted p value |

**See attached xls file for complete table**

**Supplemental Table S3. Top 20 Signature Genes Expressed by Each of the Major Cell Populations in the Human Small Airway Epithelium of Healthy Nonsmokers[1]**

| Categories | Basal | Intermediate | Club | Mucous | Ciliated |
|---|---|---|---|---|---|
| Defense[2] | | | | | |
| against pathogens, particulates | | | C3, LCN2, AGR2, CXCL17, CXCL1 | TFF3, MUC5AC, BPIFB1, SCGB1A1, MUC5B, LYZ | |
| against toxins | MGST1, FMO2 | | MGST1, ALDH1A1 | GALNT7, | |
| antiproteases | SPINT2 | SLPI | SLPI, WFDC2, SERPINB3 | WFDC2 | |
| barrier function | PERP, CLDN1 | | | CEACAM6 | |
| Proteases | | | PRSS23, CTSC | CAPN8 | |
| Cytoskeleton | KRT15, HSPB1, KRT5 | KRT19, KRT5 | KRT7, KRT19 | | |
| Protein synthesis | RPLP1, RPL32, RPL31, RPL34, RPL35, RPL7A | RPS4X, RPL3, RPL10A, RPS18, RPS24, RPL12, RPL7, RPS6, RPL5, RPS8, RPS2, RPS3A, RPL4 | | RRBP1 | |
| Growth factors | ADIRF, IL33 | IL33 | TNFSF10 | | |
| Transcription factors and regulation | | EPAS1 | ELF3 | XBP1, CREB3L1 | FHAD1 |
| Receptors | RACK1 | | ALCAM | PIGR, ADRA2A | CDHR3 |
| Maintenance of ionic balance | | | | SLC31A1 | CAPS |
| Cell respiration | MT-CO3, ATP5G2 | | | | |
| Calcium regulation | | S100A2 | | S100P | |
| Metalloprotein | | | CP | | |
| Proliferation | | | | MSMB, SCGB3A1 | |
| Signal transduction | | | | TSPAN8 | |
| Ciliary architecture | | | | | DNAAF1, DNAH12, RSPH1, CFAP43, TPPP3, SPAG17, CFAP157, DHAH5, CFAP45, CETN2, SNTN |
| Protein folding | | | | | HSP90AA1 |
| Unknown | MIR205HG | MIR205HG | FAM3D, CYP2B7P | | LRRIQ1, ERICH3, CCDC170, C20orf85, CCDC146 |

[1] The signature genes, ordered by p values (from smallest to 0.05), for each cell population were generated by comparing cells from each cell population with all other cells using Seurat "FindAllMarkers" function. The signature genes were expressed in >10% of the cells in the corresponding cell populations, and the average expression levels of the expressing cells in the corresponding cell population *vs* all other expressing cells were >0.25 (log). Bonferroni corrected $p<0.05$ was used as the cutoff.

[2] Defense-related genes include those against pathogens, particulates, toxins, proteases and barrier function.

**Supplemental Table S4. Top 20 Signature Genes Expressed by Each of the Minor Cell Populations in the Human Small Airway Epithelium of Healthy Nonsmokers[1]**

| Categories | Ionocyte | Neuroendocrine | T cell | Mast | Antigen presenting | NCL$^{high}$ |
|---|---|---|---|---|---|---|
| Cytokine | RARRES2, IGF1 | | CCL5, IL32 | | | |
| Cell surface molecules | | | CD2, PTPRC, TRBC, CD52, B2M, HLA-B, HLA-C, HLA-E, TRAC, IL7R, HLA-A, CD3D, CD3E, TRBC1, CD3G | KIT, CD52 | HLA-DRA, HLA-DRB1, CD74, HLA-DPB1, HLA-DPA1, HLA-DQB1, HLA-DQA1, HLA-DMB, HLA-DRB5, HLA-DQB2 | |
| Protein secretion | SEC11C | RTN1, CHGA | | SRGN, LAPTM5 | | |
| Protein degradation | | UBB | | | | HSP90AB1 |
| Cytoskeleton | | TUBA4A, MAP1B, TUBA1A | TMSB4X, EVL | VIM, CAPG | TMSB10 | |
| Signal transduction | STAP1, CLNK | CALM1, CALM3, GNG13, FSTL5, CALM2, GNAL, RIC8B | | TYROBP | | |
| Fat metabolism | | | | APOE | | |
| Transcription factors | ASCL3, TFCP2L1, FOXI1 | | | | | |
| Receptors | ADGRF5 | | | | | |
| Maintenance of ionic balance | ATP6V1G3, CLCNKB, ITPR2, GABRB2, ATP6V0B | ATP1B1, STOML3 | | | | |
| Defense | | | | | | |
| against pathogens, particulates | | | | | LYZ | |
| against toxins | DGKI | | | | CYBB | |
| against protease | | | | | CST3 | |

**Supplemental Table S4. Top 20 Signature Genes Expressed by Each of the Minor Cell Populations in the Human Small Airway Epithelium of Healthy Nonsmokers[1] (cont., page 2)**

| Categories | Ionocyte | Neuroendocrine | T cell | Mast | Antigen presenting | NCL$^{high}$ |
|---|---|---|---|---|---|---|
| Glucose and insulin homeostasis | APLP2 | | | | | |
| Mitosis | HEPACAM2 | | | | | |
| Development | SEMA3C | | | | | |
| Proliferation | | | | | AIF1, LST1 | |
| Lysosomal targeting | | | | | PSAP | |
| Cellular ion regulation | | | | | FTL | |
| Cellular energy homeostasis | | CKB | | | | |
| Chromatin regulation | | | | | | NCL, HMGB2 |
| Unknown | LINC01187, TMEM61 | CALM1P1, CALM1P2, S100A5, HSP90AB3P | GIMAP7 | VWA5A, SLC45A3, DTNBP1 | HLA-DRB6, MNDA | PTMAP2, PTMAP5, HSP90AA2P, HSP90AA5P, RPSAP55, RPSAP28, NPM1P4, HSP90AB3P, RP11-538P18.1, NPM1P8, RP11-253E3.1, CTA-351J1.1, NPM1P43, RPL37AP8, AB019441.29, HSP90AA6P, FAUP1 |

[1] The signature genes, ordered by p values (from smallest to 0.05), for each cell population were generated by comparing cells from each cell population with all other cells using Seurat "FindAllMarkers" function. The signature genes-were expressed in >10% of the cells in the corresponding cell populations, and the average expression levels of the expressing cells in the corresponding cell population *vs* all other expressing cells-were >0.25 (log). Bonferroni corrected p<0.05 was used as the cutoff.

[2] Defense-related genes include those against pathogens, particulates, toxins and proteases.

## Supplemental Table S5. Differentially Expressed Genes in Nonsmokers *vs* Smokers in the Major Human Small Airway Epithelial Cell Populations

| Basal | | | | Intermediate | | | | Club | | | | Mucous | | | | Ciliated | | | |
|---|---|---|---|---|---|---|---|---|---|---|---|---|---|---|---|---|---|---|---|
| Down-regulated genes | | Up-regulated genes | | Down-regulated genes | | Up-regulated genes | | Down-regulated genes | | Up-regulated genes | | Down-regulated genes | | Up-regulated genes | | Down-regulated genes | | Up-regulated genes | |
| Gene symbol | Adjusted p value[1] | Gene symbol | Adjusted p value | Gene symbol | Adjusted p value | Gene symbol | Adjusted p value | Gene symbol | Adjusted p value | Gene symbol | Adjusted p value | Gene symbol | Adjusted p value | Gene symbol | Adjusted p value | Gene symbol | Adjusted p value | Gene symbol | Adjusted p value |

[1] Adjusted p value means Bonferroni corrected p value and that down- and up-regulation is in smokers compared to nonsmokers

**See attached xls file for complete table**

**Supplemental Table S6. Categories of the Top 25[1] Differentially Expressed Genes in Nonsmokers *vs* Smokers in the Major Human Small Airway Epithelial Cell Populations**

| | BC | | Intermediate | | Club | | Mucous | | Ciliated | |
|---|---|---|---|---|---|---|---|---|---|---|
| **Categories** | **Down[2]** | **Up[2]** | **Down[2]** | **Up[2]** | **Down[2]** | **Up[2]** | **Down[2]** | **Up[2]** | **Down[2]** | **Up[2]** |
| Defense against pathogens, particulates | SCGB1A1* | | SCGB1A1*<br>MUC5B*<br>C3*<br>LCN2<br>LTF*<br>LYPD2*<br>TNFAIP2<br>B2M | | C3*<br>MUC5B*<br>LCN2<br>LTF*<br>SCGB1A1*<br>LYPD2*<br>TNFAIP2<br>SAA1*<br>FCGBP*<br>CXCL6*<br>CCL20* | | SCGB1A1*<br>MUC5B*<br>LYPD2*<br>FCGBP*<br>ITLN1<br>HLA-B | BPIFB1*<br>MUC5AC<br>AGR2*<br>B3GNT6*<br>CXCL17<br>PTMA | SCGB1A1*<br>SAA1*<br>HLA-DRA<br>ALCAM<br>CXCL1<br>B2M | MUC5AC |
| against toxins | | ALDH3A1*<br>TXNIP | GDA* | ALDH3A1*<br>CYP1B1*<br>TXNIP<br>PRDX1* | | ALDH3A1*<br>CYP1B1*<br>NQO1*<br>CYP26A1*<br>PRDX1*<br>TXNIP<br>AKR1C2* | CY4B1<br>ODC1 | ALDH3A1*<br>CYP1B1* | HE3ST1<br>CYP4B1 | ALDH3A1*<br>NQO1*<br>AKR1C2*<br>AKR1C1*<br>ADH7*<br>GSTA2*<br>PRDX1*<br>GPX2*<br>ABHD2*<br>AKR1B10*<br>CES1<br>TKT*<br>TXNRD1*<br>TALDO1* |
| antiproteases | SLPI | | SLPI | | SLPI<br>PI3* | SERPINB1 | | WFDC2 | SLPI | |
| barrier functions | | PERP | | PERP | | PERP | | CEACAM5*<br>CEACAM6* | | |
| Proteases | | | | | | | KLK11<br>KLK10 | | | |
| Anti-apoptosis | | IER3 | | IER3 | | IER3 | | | | |
| Cytoskeleton | | KRT17* | RHOV | KRT17* | RHOV | KRT18 | | EZR | | KRT8 |

**Supplemental Table S6. Categories of the Top 25 Differentially Expressed Genes in Nonsmokers *vs* Smokers in the Major Human Small Airway Epithelial Cell Populations[1] (cont., page 2)**

| | BC | | Intermediate | | Club | | Mucous | | Ciliated | |
|---|---|---|---|---|---|---|---|---|---|---|
| **Categories** | **Down[2]** | **Up[2]** | **Down[2]** | **Up[2]** | **Down[2]** | **Up[2]** | **Down[2]** | **Up[2]** | **Down[2]** | **Up[2]** |
| | | KRT15<br>KRT5<br>TPM1 | | KRT18 | | | | ACTG1<br>KRT18 | | |
| Protein synthesis | RPS29 | RPS3A<br>RPL3<br>RPS2<br>RPLP0 | | RPS18<br>RPS2<br>RPLP0<br>RPL18A | | RPS18<br>RPLP0<br>RPS2 | | RPS6 | RPLP1<br>RPS29 | |
| Growth factors | | NTS<br>CTGF* | | NTS | | NTS | | | | SPP1* |
| Transcription factors and regulation | | JUN<br>TSC22D1<br>FOS | | JUN<br>ATF3<br>TSC22D1 | | JUN | PAX5<br>HES1 | JUN | | |
| Receptors | | F3 | PIGR | F3 | PIGR | | PILRB<br>ADRA2A**<br>PTGFR | CD24<br>CD55 | PIGR<br>ITGA2<br>CD74 | |
| Maintenance of ionic balance | CLCA2 | AQP5 | | | | | | | PIEZO2 | S100A10* |
| Cell respiration | MT-CO3<br>MT-ND3 | | MT-ND3 | | | MT-CO1<br>MT-CO2 | MT-ND2<br>MT-ND3 | | MT-ND3<br>MT-ATP6 | |
| Cellular ion regulation | | FTL* | | | MT3 | FTH1*<br>FTL* | | S100A6<br>FTL* | | FTL*<br>FTH1* |
| Metalloprotein | | | CP | | CP | | | | | |
| Proliferation | SCGB3A1 | | SCGB3A1 | H19* | SCGB3A1<br>TMEM45A* | H19* | SCGB3A1 | TPT1 | SCGB3A1 | |
| Signaling transduction | | CYR61<br>SDPR*<br>THSD4 | | SDPR*<br>TSPAN1*<br>PPP1R15A | | SFRP2* | | TSPAN1* | | |
| Ciliary architecture | RSPH1 | | | | | | | | | TUBA1A* |
| Protein folding | | | | | HSP90AA1 | | | | | |
| Extracellular matrix | | | | LAMB3 | | | | | | |
| Cell migration | | | MALAT1 | IER2,<br>SNHG5 | MALAT1 | ANXA2 | | | MALAT1 | |
| Cellular membrane composition | | | | | | | | | | |
| **Stem cell marker** | | | | | PROM1 | | | | | |
| Neuro-regulation | | MT-RNR2 | | MT-RNR2 | | MT-RNR2 | | MT-RNR2 | SLITRK6 | |

**Supplemental Table S6. Categories of the Top 25 Differentially Expressed Genes in Nonsmokers *vs* Smokers in the Major Human Small Airway Epithelial Cell Populations[1] (cont., page 3)**

| | BC | | Intermediate | | Club | | Mucous | | Ciliated | |
|---|---|---|---|---|---|---|---|---|---|---|
| **Categories** | **Down[2]** | **Up[2]** | **Down[2]** | **Up[2]** | **Down[2]** | **Up[2]** | **Down[2]** | **Up[2]** | **Down[2]** | **Up[2]** |
| | | SLITRK6 | | | | | | | | |
| Muscle contraction | | | | | | | TNNT3** | | | |
| Unknown | MTRNR2L1 | | SAA2*<br>BICDL2 | | MTRNR2L3<br>SAA2*<br>ALPL | LY6D | CTD-2531D15.4<br>SNHG25<br>ALPL<br>MTRNR2L3<br>MT-TV<br>MT-TP | PSCA<br>NEAT1 | MTRNR2L1<br>ABCA13*<br>MTRNR2L3<br>OSBPL6*<br>EPB41L2* | TMEM190*<br>PSCA<br>LDLRAD1<br>MUCL1* |

*Differentially expressed genes identified in the total small airway epithelium

**Differentially expressed genes identified in the total small airway epithelium with opposite direction

[1] Pseudogenes were excluded. Only 9 and 18 gene were down-regulated in BC and intermediate cells, respectively.

[2] Up or down-regulated genes in smokers compared to nonsmokers. These genes were expressed in >10% of the cells in the corresponding cell populations in both nonsmokers and smokers, and the average expression levels of the expressing cells in the corresponding cell population in nonsmokers *vs* smokers were >0.25 (up-regulated genes) or <-0.25 (down-regulated genes). Bonferroni corrected p<0.05 was used as the cutoff.

**Supplemental Table S7. Cell Numbers of Ciliated Cell Sub-populations in Each Individual**

| Sub-populations | Nonsmoker-1 | Nonsmoker-2 | Nonsmoker-3 | Smoker-1 | Smoker-2 | Smoker-3 |
|---|---|---|---|---|---|---|
| 1 | 109 | 0 | 9 | 5 | 0 | 3 |
| 2 | 102 | 28 | 96 | 109 | 45 | 48 |
| 3 | 4 | 3 | 2 | 117 | 56 | 24 |
| 4 | 6 | 29 | 111 | 31 | 8 | 25 |

**Supplemental Table S8. Smoking-induced Changes in Human Small Airway Cell-specific Expression of Genes-related to Lung Diseases**

| | BC | | Intermediate | | Club | | Mucous | | Ciliated | | Ionocytes | | T cells | | Mast | |
|---|---|---|---|---|---|---|---|---|---|---|---|---|---|---|---|---|
| **Lung diseases** | **Down[1]** | **Up[1]** | **Down[1]** | **Up[1]** | **Down[1]** | **Up[1]** | **Down[1]** | **Up[1]** | **Down[1]** | **Up[1]** | **Down[1]** | **Up[1]** | **Down[1]** | **Up[1]** | **Down[1]** | **Up[1]** |
| Monogenic lung disorders | RSPH1* | | | | | TGFBR2 | | | OFD1* | C21orf59* | | SFTPB* | | | EFEMP2* | |
| a | | THSD4* | | THSD4<br>PID1 | | TGFB2 | | | | | | | | | | |
| IPF | | | MUC5B* | CDKN1A | MUC5B* | CDKN1A | MUC5B* | | | | | | MUC5B* | | | |
| Lung cancers | | TP63* | B2M* | SLC34A2<br>EGFR<br>MCL1<br>PTP4A1<br>PHF3<br>MYC<br>KLF5<br>TPM3<br>NFE2L2 | B2M<br>FGFR3* | MDM2 | | EZR*<br>TPM3* | B2M*<br>CD74* | EZR* | | | B2M<br>ZFP36L1 | | TP73*<br>DOT1L*<br>PDE4DIP*<br>MAP2K1* | |

[1] Up or down-regulated genes in smokers compared to nonsmokers. Bonferroni corrected p<0.05 was used as the cutoff.

* For those genes, the average expression levels of the expressing cells in the corresponding cell population in nonsmokers *vs* smokers were >0.25 (up-regulated genes) or <-0.25 (down-regulated genes)

**Supplemental Table S9. Quality Control for the Single-cell RNA-sequencing Data in Each Individual[1]**

| Phenotype | Total reads | Mean reads/cell | Number of gene detected | Median genes/cell | Median UMI count/cell |
|---|---|---|---|---|---|
| Nonsmoker-1 | 112,793,786 | 91,405 | 29,816 | 736 | 1,390 |
| Nonsmoker-2 | 131,807,942 | 107,686 | 31,800 | 896 | 1,517 |
| Nonsmoker-3 | 67,651,975 | 29,842 | 30,250 | 769 | 1,259 |
| Smoker-1 | 113,288,982 | 43,690 | 31,544 | 816 | 1,393 |
| Smoker-2 | 47,156,371 | 23,472 | 28,539 | 830 | 1,465 |
| Smoker-3 | 128,445,800 | 54,082 | 31,840 | 727 | 1,247 |

[1]Table reflects quality control data after applying filters during processing; UMI = unique molecular identifiers.

**Supplemental Table S10 . Cell Numbers of Different Cell Populations in Each Individual**

| Cell type | Nonsmoker-1 | Nonsmoker-2 | Nonsmoker-3 | Smoker-1 | Smoker-2 | Smoker-3 |
|---|---|---|---|---|---|---|
| Basal | 109 | 122 | 206 | 395 | 1112 | 417 |
| Intermediate | 266 | 248 | 577 | 513 | 444 | 590 |
| Club | 405 | 252 | 586 | 213 | 70 | 253 |
| Mucous | 18 | 141 | 11 | 542 | 153 | 255 |
| Ciliated | 221 | 60 | 218 | 262 | 109 | 100 |
| Ionocytes | 20 | 11 | 54 | 82 | 32 | 40 |
| Neuroendocrine | 0 | 0 | 146 | 0 | 0 | 0 |
| T cell | 171 | 244 | 436 | 418 | 45 | 651 |
| Antigen-presenting | 22 | 21 | 33 | 76 | 31 | 24 |
| Mast | 2 | 4 | 0 | 91 | 13 | 44 |
| $NCL^{high}$ | 0 | 121 | 0 | 1 | 0 | 1 |

**Supplemental Table S11. Effect of Gender *vs* Smoking on Small Airway Epithelium Gene Expression[1]**

| | Microarray[2] | | RNA-Seq[3] | |
|---|---|---|---|---|
| **Comparison[1]** | **n of subjects[2]** | **n of genes[4]** | **n of subjects** | **n of genes[4]** |
| Nonsmoker M *vs* nonsmoker F | 38 M *vs* 22 F | 30 | 10 M *vs* 10 F | 42 |
| Smoker M *vs* smoker F | 53 M *vs* 20 F | 23 | 20 M *vs* 3 F | 16 |
| M *vs* F | 91 M *vs* 42 F | 40 | 30 M *vs* 13 F | 68 |
| Smoker *vs* nonsmoker | 73 S *vs* 63 NS | 3,408 | 23 S *vs* 20 NS | 2,454 |

[1] Analysis of previously acquired datasets; M = males, F = females, NS = healthy nonsmokers, S = asymptomatic smokers.

[2] Small airway epithelial samples processed on Affymetrix HG-U133 Plus 2.0 microarrays (Affymtrix); previously published (GEO accession # 77658).

[3] Small airway epithelial samples processed on Illumina Hi Seq 2500 (Illumina); a subset of the samples has been previously published (GEO accession # 92661).

[4] N of genes differentially expressed when comparing the groups on a genome-wide basis [in microarray: n=14,465 genes (present in at least 20% of the samples in each group , one probe per gene, chosen based on Affymetrix specificity and sensitivity scores); in RNA-Seq: n=16,140 genes (FPKM >0.125)]; p value corrected for multiple tests (Benjamini-Hochberg) <0.05 considered significant.

## Supplemental Figure Legends

**Supplemental Figure S1.** Violin plots of the expression of TP63 and MKI67 in the cells populations identified in Figure 1. The violin plots were constructed using imputed data. **A.** TP63. **B.** MKI67.

**Supplemental Figure S2.** Human small airway ionocyte transcriptome and comparison with the ionocytes from large airways. **A.** Gene Ontology (https://david.ncifcrf.gov/) analysis of the signature genes of human small ionocytes. **B.** Venn diagram of signature genes of ionocytes in human small airways *vs* large airways. The large airway ionocyte data is from Montoro et al [18]. Shown are the number of genes uniquely enriched in human large and/or small airways. Examples of the signature genes are indicated. **C.** Violin plot of POSTN expression in the cell populations from nonsmoker human small airway epithelium. The violin plots were constructed using imputed data. SAE = small airway epithelium, LAE = large airway epithelium.

**Supplemental Figure S3.** Expression of genes-associated with monogenic lung disorders in the cell populations of the healthy human small airway epithelium. **A-F.** Genes associated with monogenic lung disorders divided by different categories: **A, B.** Primary ciliary dyskinesia; **C.** Cystic fibrosis and other bronchiectasis; **D.** α1-antitrypsin deficiency, Birt-Hogg Duke syndrome, cutis laxa, Ehlers-Danlos syndrome, lymphangioleiomyomatosis, Loey-Diez syndrome, and Marfan syndrome; **E.** Familial fibrosis, Fibrosis and hypothyroidism, Hermansky-Pudlak syndrome, pulmonary alveolar proteinosis, surfactant deficiency; **F.** Pulmonary hypertension with arteriovenous malformations and hereditary hemorrhagic telangiectasia, pulmonary hypertension with hereditary hemorrhagic telangiectasia, pulmonary hypertension, syndromic hypoventilation, hypereosinophilic syndrome, and hyper IgE syndrome. The genes are shown on the x-axis. The identities of the cell populations are shown on the y-axis. The size of the dots represents the fractions of the expressing cells in each cell population, and the color intensity represents the average

expression level. **G-L.** Violin plots of the selected monogenic lung disorder genes in the cell populations from nonsmoker human SAE. The violin plots were constructed using imputed data. **G.** SERPINA1 (α1-antitrypsin deficiency); **H.** CFTR (cystic fibrosis); **I.** SCNN1B (bronchiectasis); **J.** RSPH9 (primary ciliary dyskinesia); **K.** DOCK8 (hyper IgE syndrome); and **L.** DTNBP1 (Hermansky-Pudlak syndrome).

**Supplemental Figure S4.** Violin plots of expression of genes associated with risk for chronic obstructive pulmonary disease (COPD) in the cell populations from healthy human small airway epithelium. The violin plots were constructed using imputed data. **A, B.** Definite COPD risk genes; **C-E.** Probable COPD risk genes. **A.** FAM13A; **B.** DSP; **C.** ARMC2; **D.** CFDP1; and **E.** TET2.

**Supplemental Figure S5.** Expression in the healthy human small airway epithelium cell populations of genes associated with risk for idiopathic pulmonary fibrosis (IPF). Genes associated with IPF are divided by 3 categories: **A.** alveolar stability and telomere length; **B.** immunity and inflammation; and **C.** common genetic variation. The gene symbols are listed on the x-axis, the identities of the cell populations are shown in y-axis. The size of the dots represents the fractions of the expressing cells in each cell population, and the color intensity represents the average expression level. **D-I.** Violin plots of genes related to risk for IPF in the cell populations from healthy human SAE. The violin plots were constructed using imputed data. **D.** MUC5B; **E.** MUC2; **F.** TGFB1; **G.** CDKN1A; **H.** HSPA1L; and **I.** HLA-DRB1.

**Supplemental Figure S6.** Violin plots of examples of small airway epithelium cell-specific expression of "driver" genes which participate in the development of lung cancer. The violin plots were constructed using imputed data. **A.** EGFR; **B.** KRAS; **C.** MET; **D.** TP53; **E.** KIF5B; and **F.** SOX2.

**Supplemental Figure S7.** Cigarette smoking-associated dysregulated genes in the minor cells

populations from human small airway epithelium of nonsmokers *vs.* smokers . Volcano plots show the down-regulated (left) and up-regulated genes (right) in smokers in ionocytes, T cells, mast cells, and antigen presenting cells. Y-axis represents the negative p value (log) and the x-axis represents the fold-change (log). The cutoff is shown as dotted lines. Fold-change (log) >0.25 for up-regulated genes or < –0.25 for down-regulated genes, p value <0.05 with Bonferroni correction. NS = nonsmokers, S = smokers.

**Supplemental Figure S8.** Impact of cigarette smoking on gene expression in the ciliated cell sub-populations of the human SAE. **A.** Unsupervised t-SNE clustering identifies 4 unique ciliated cell sub-populatFhions in nonsmokers (left) *vs* smokers (right). **B.** Fractions of ciliated cell sub-populations in nonsmokers *vs* smokers in each individual, nonsmoker (n=3) *vs* smoker (n=3). **C.** Dot plots of gene expressions in the ciliated cell sub-populations. The ciliated cell sub-populations are shown on the y-axis, and the gene symbol and detailed categories of the genes are shown on the x-axis. The size of the dots represents the fraction of the expressing cells in each cell population. The color represents the average gene expression in positive cells.

**Supplemental Figure S9.** Single-cell RNA sequencing identifies 11 unique cell populations from human SAE of 6 individuals. **A.** t-SNE plots of the single cells from each individuals. **B.** t-SNE plots of the single cells from 4 different technical batches. Batch 1 – nonsmoker 1 + smoker 1; batch 2 – nonsmoker 2; batch 3 – nonsmoker 3 + smoker 2; batch 4 – smoker 3. NS = nonsmokers, S = smokers.

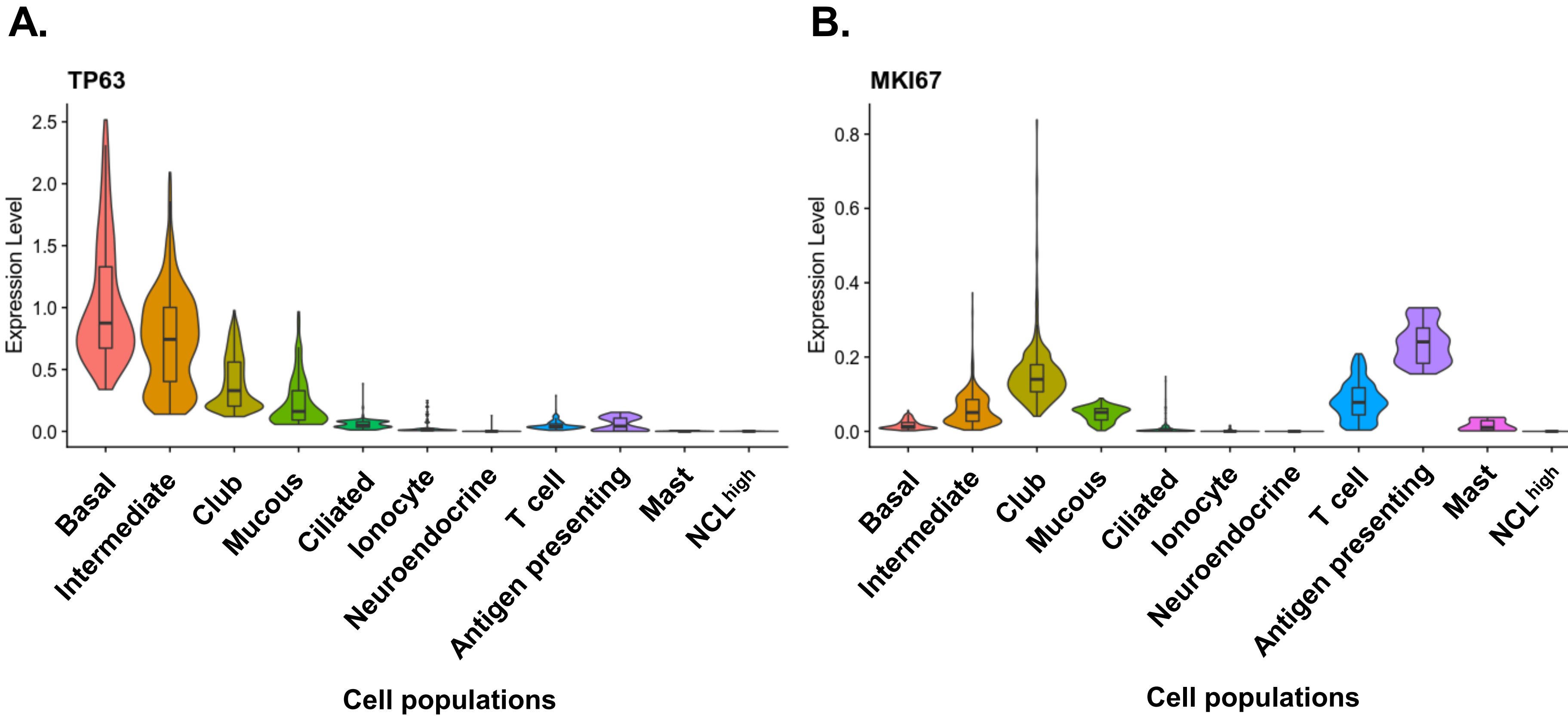


Supplemental Figure 1

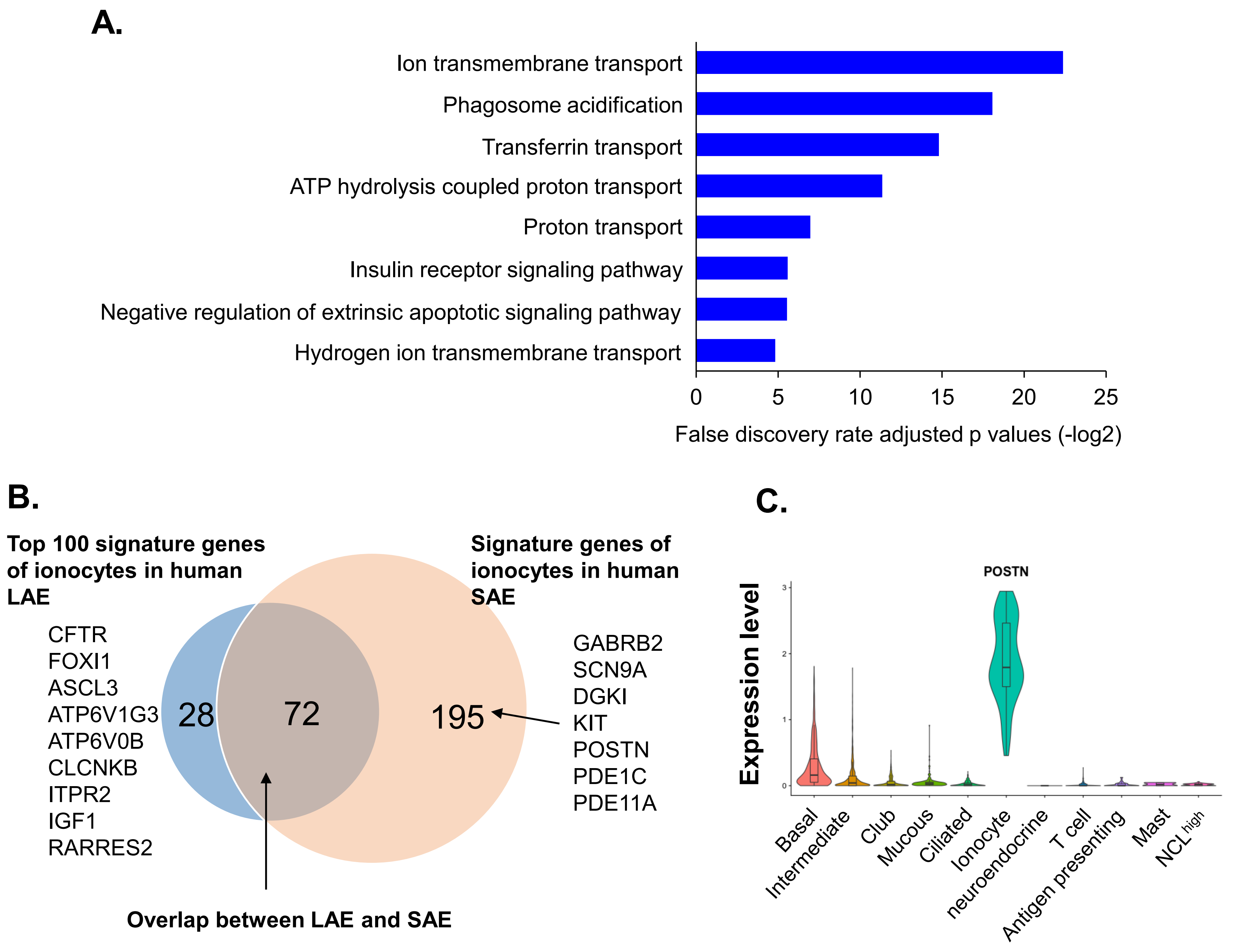


Supplemental Figure 2

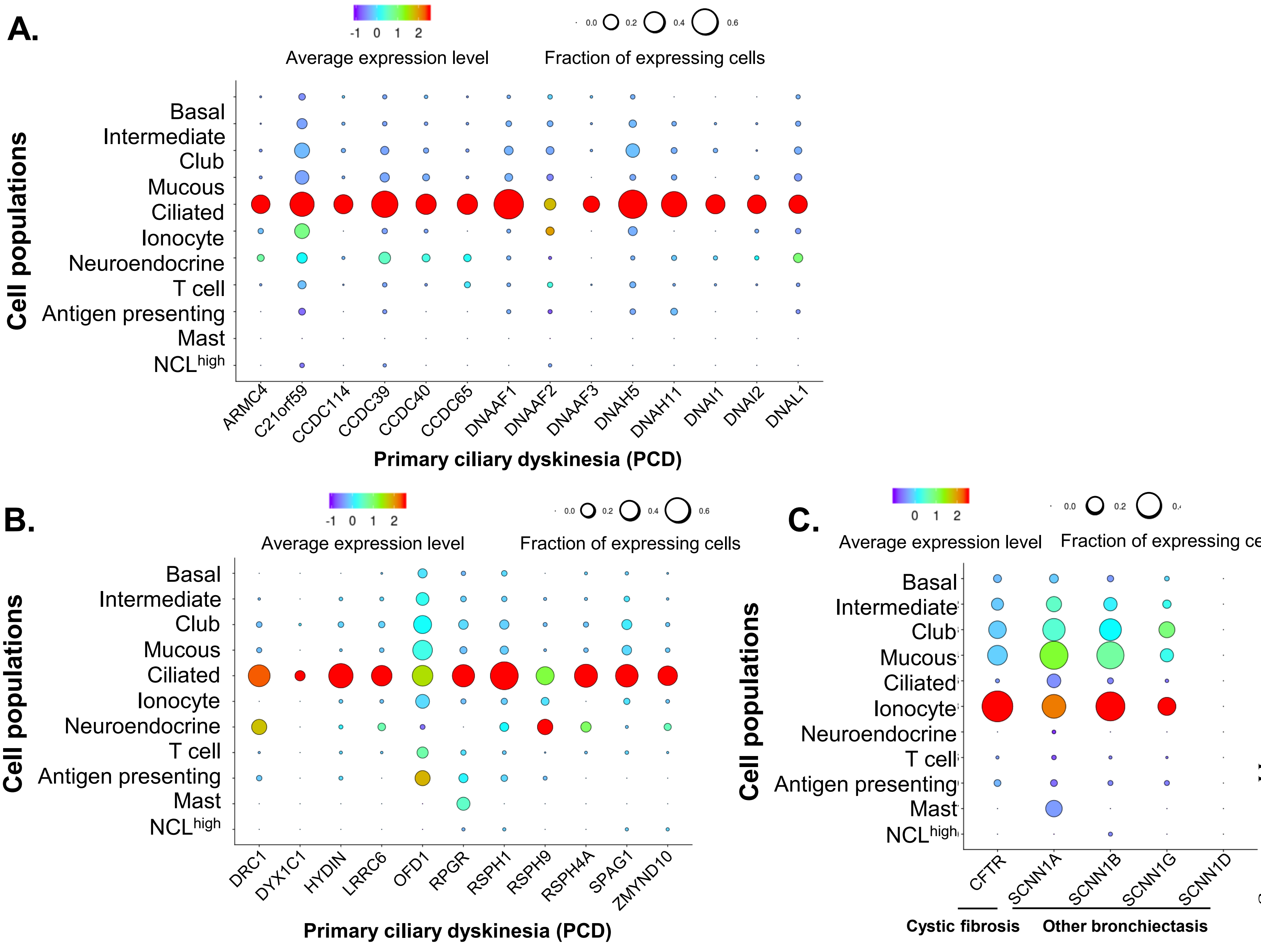


Supplemental Figure 3A

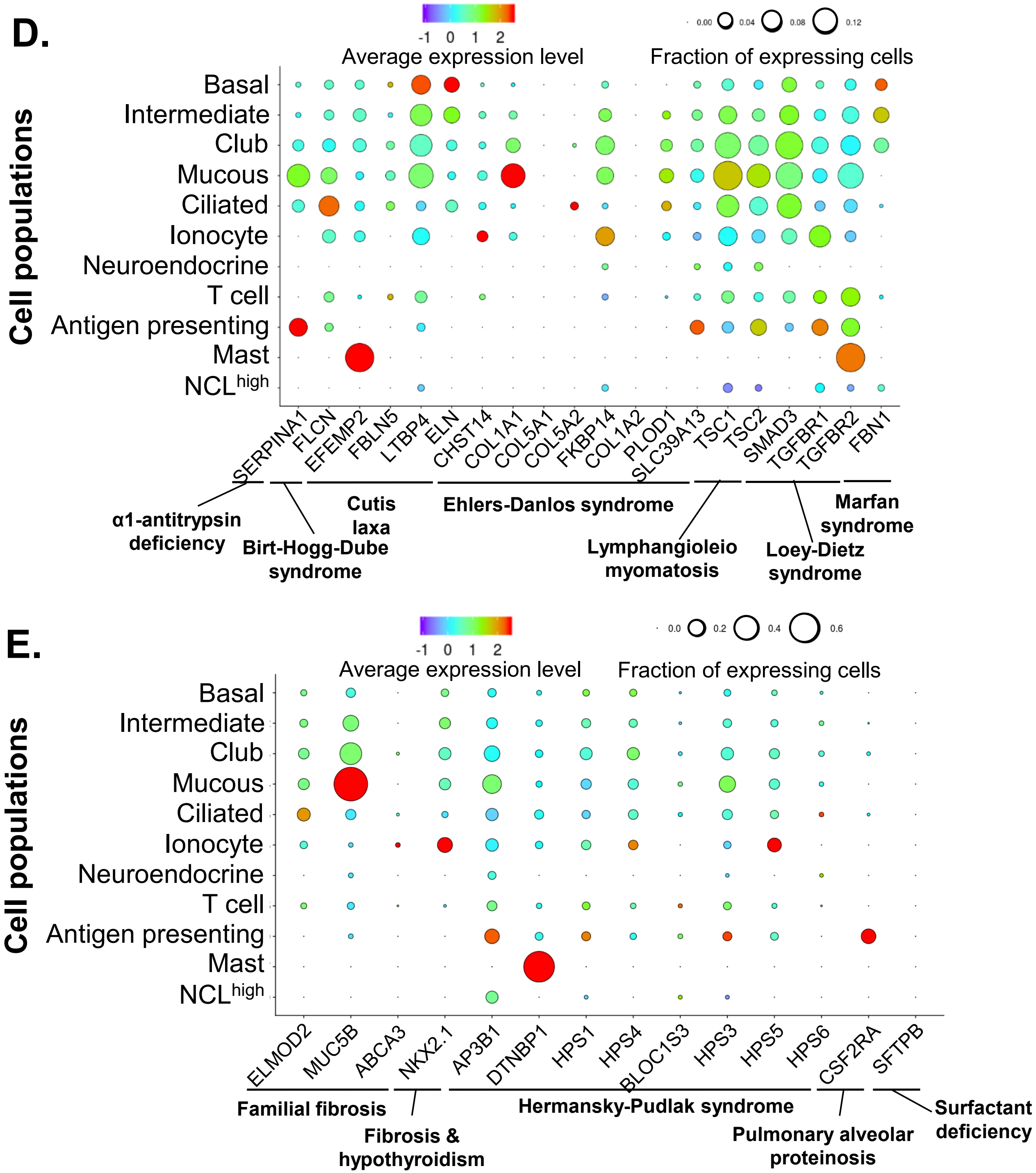


Supplemental Figure 3B

F.

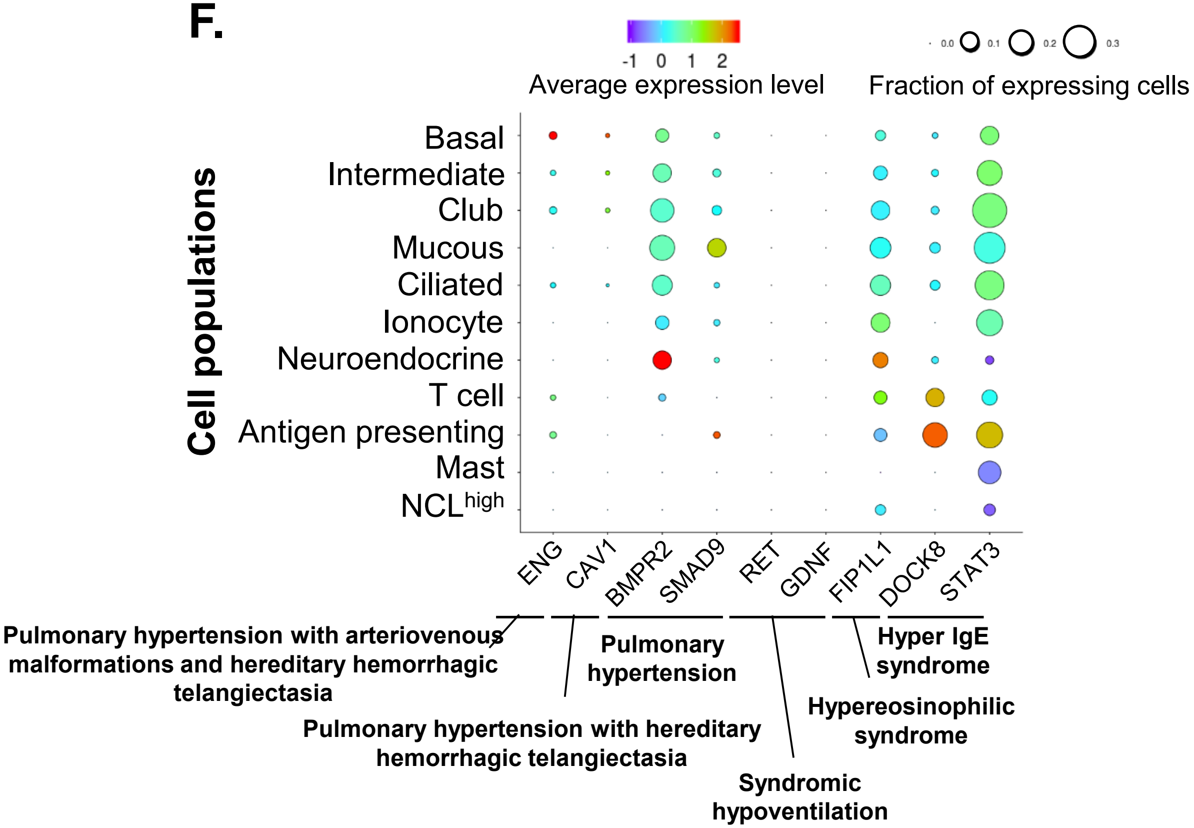


Supplemental Figure 3C

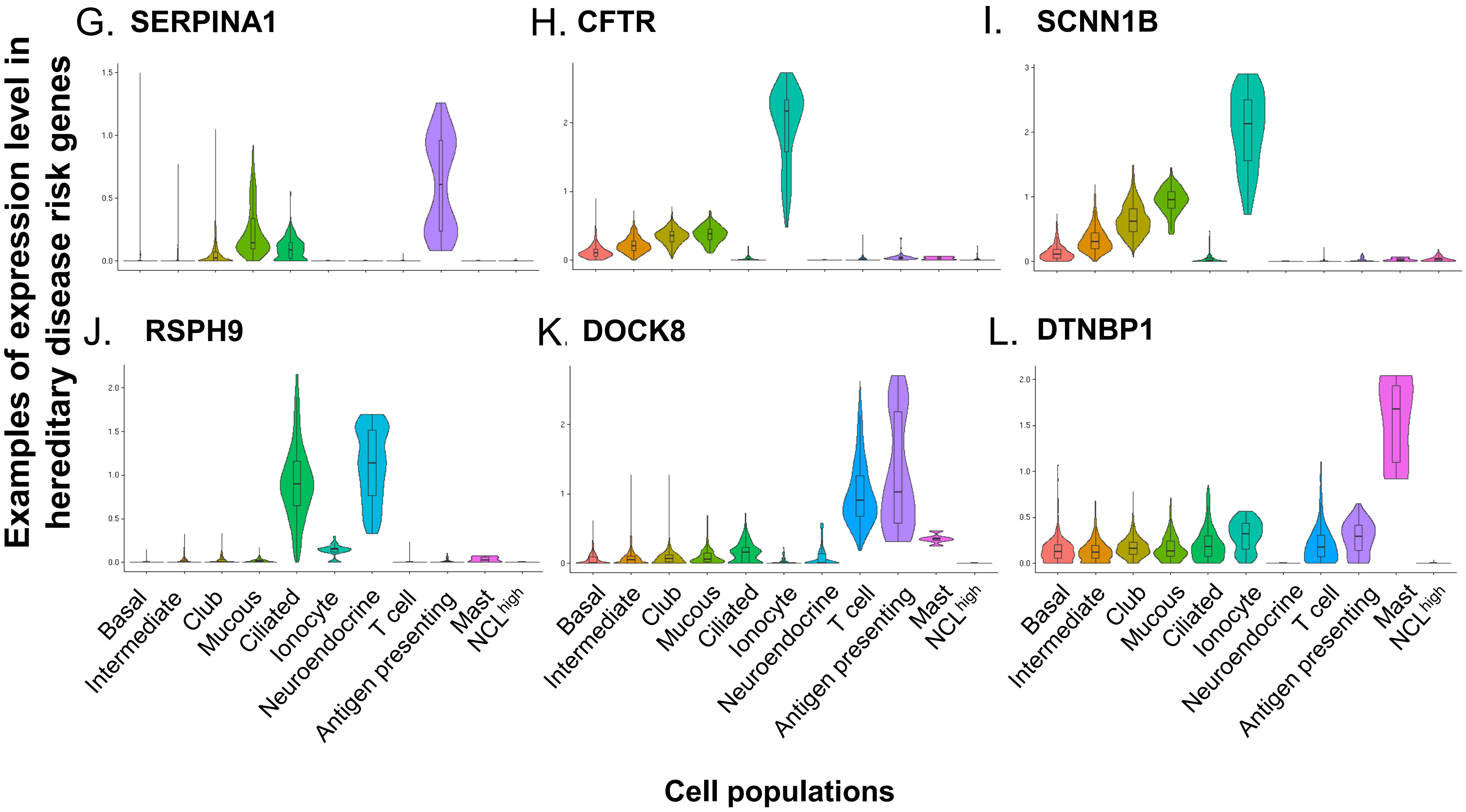


Supplemental Figure 3D

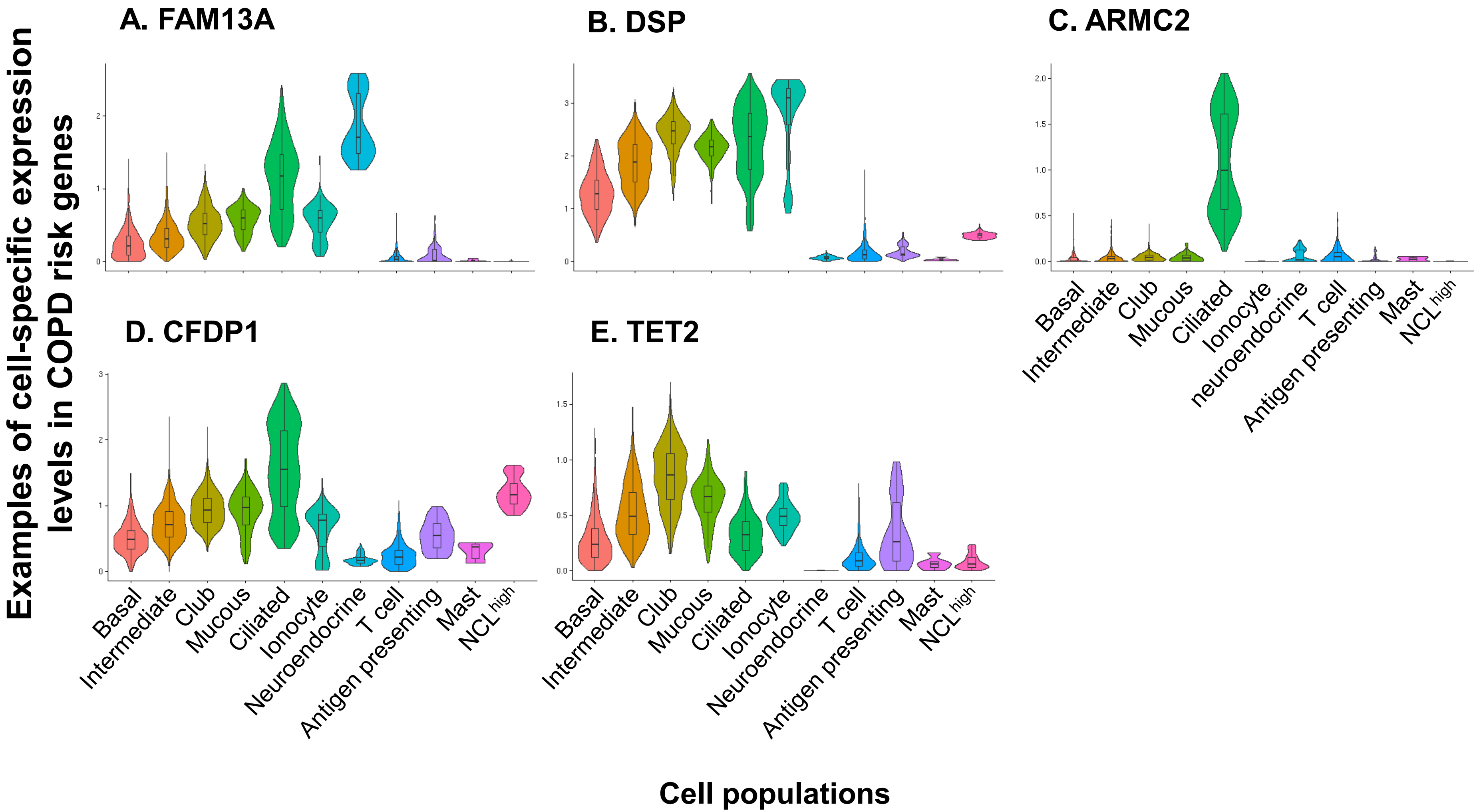


Supplemental Figure 4

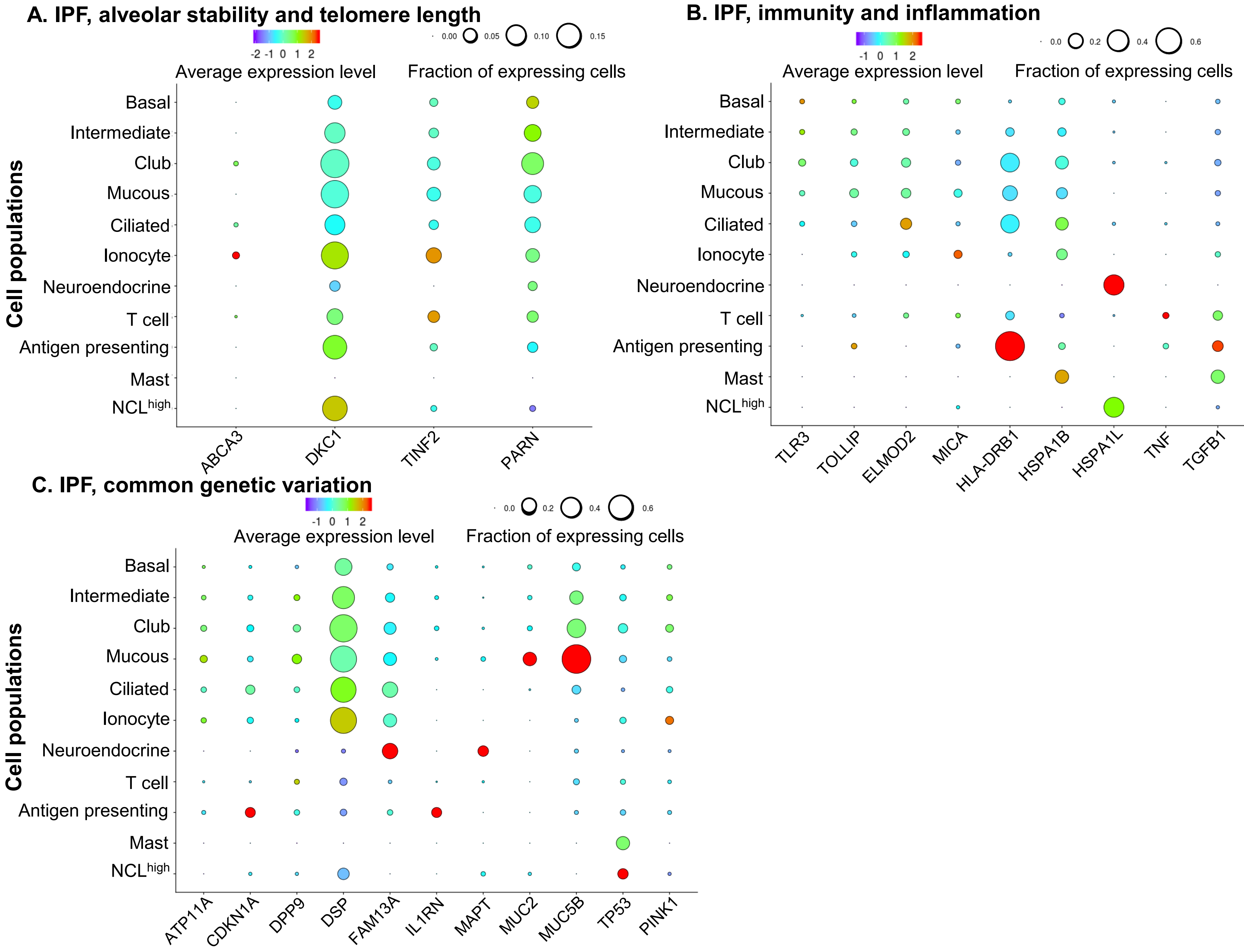


Supplemental Figure 5

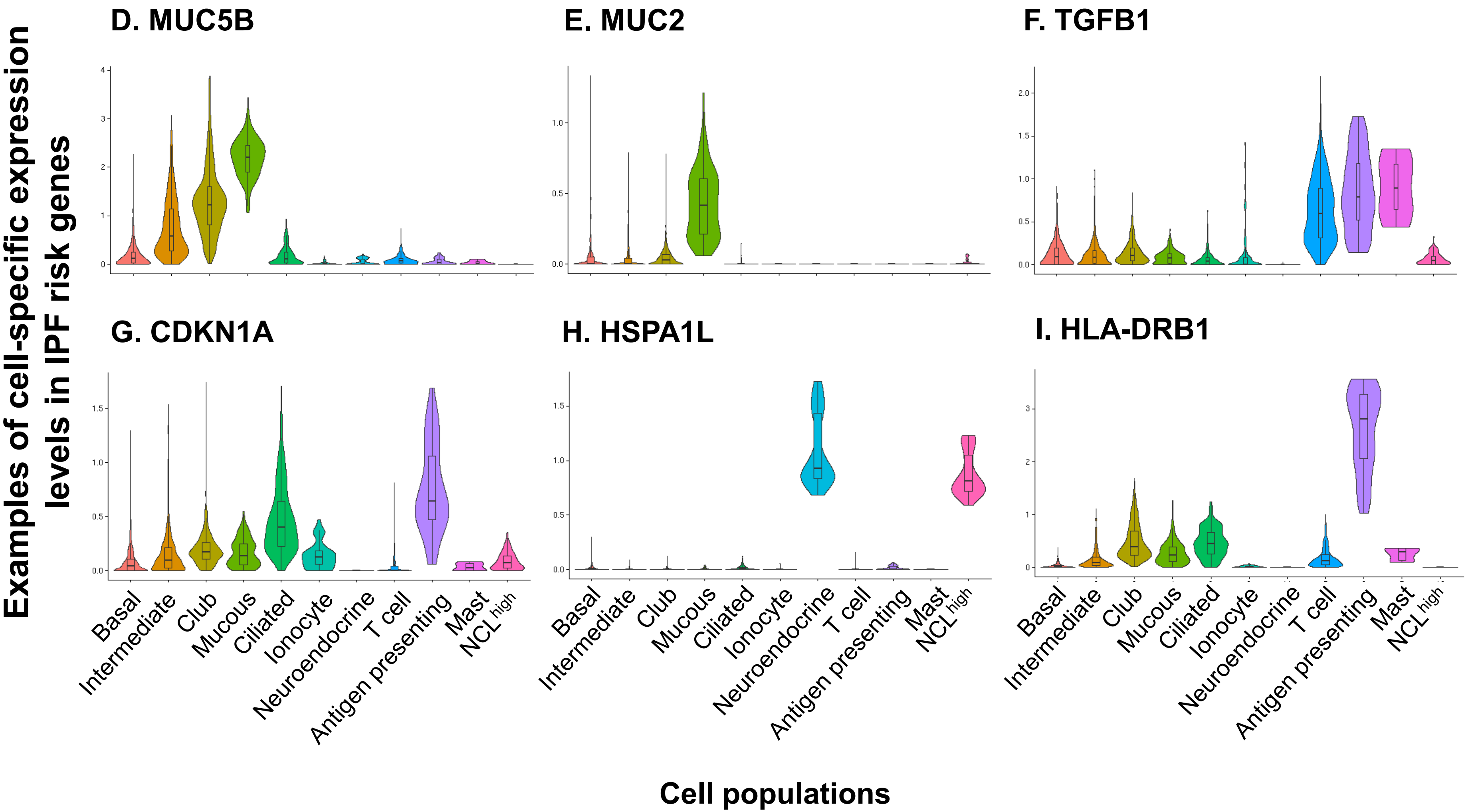


Supplemental Figure 5

Examples of cell-specific expression levels of "driver" genes that if mutated participate in the development of lung cancer

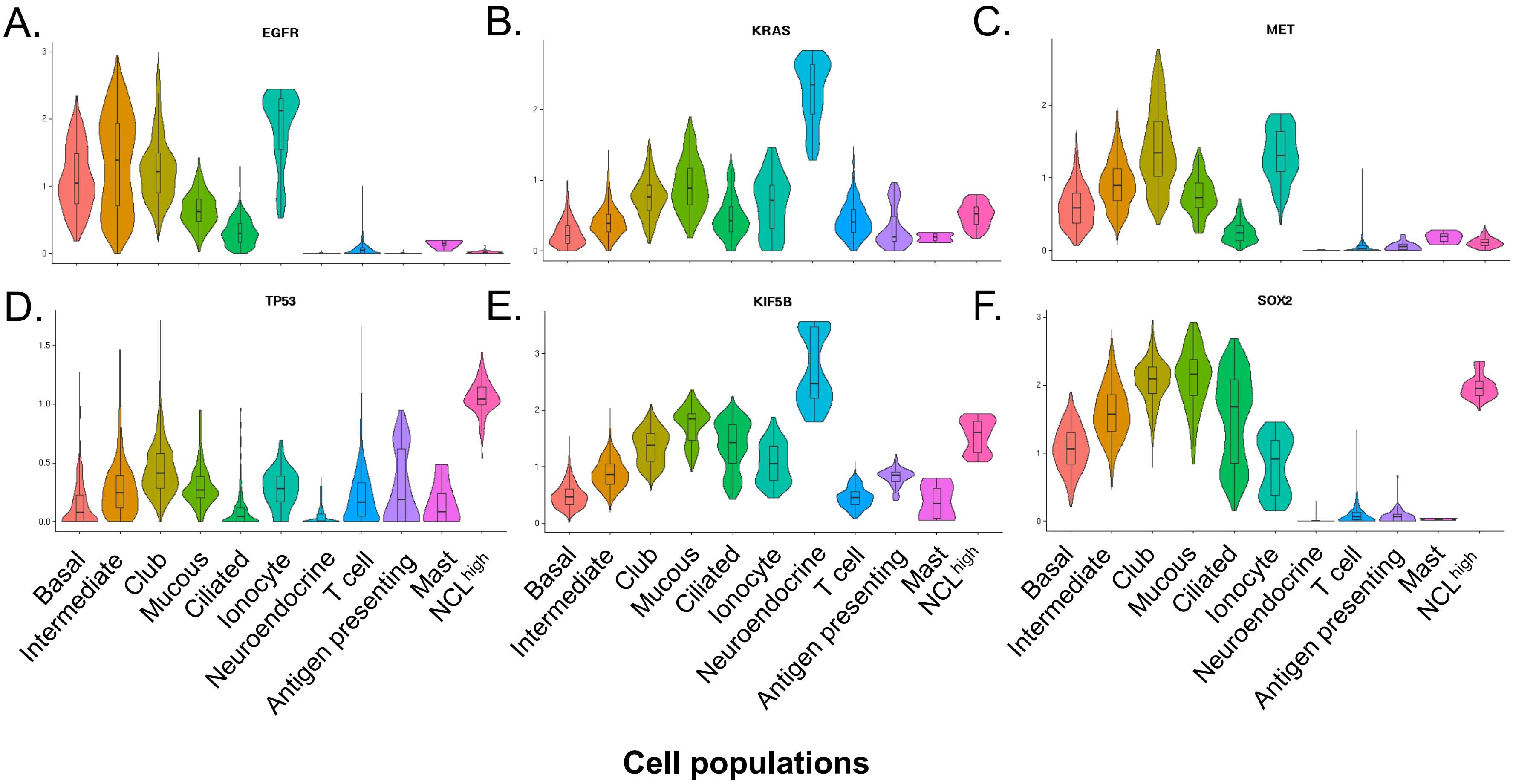


Supplemental Figure 6

**Supplemental Figure 7**

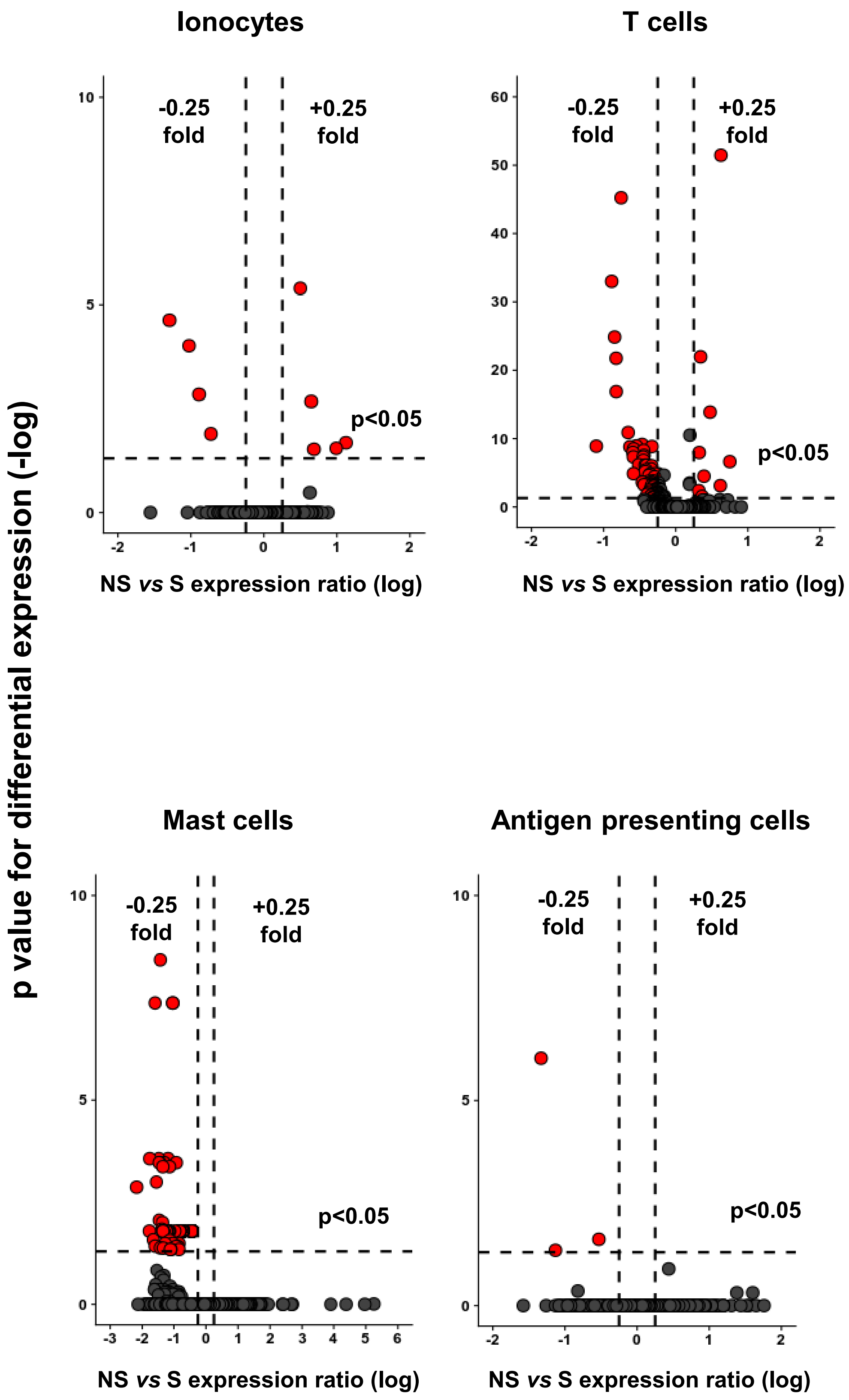

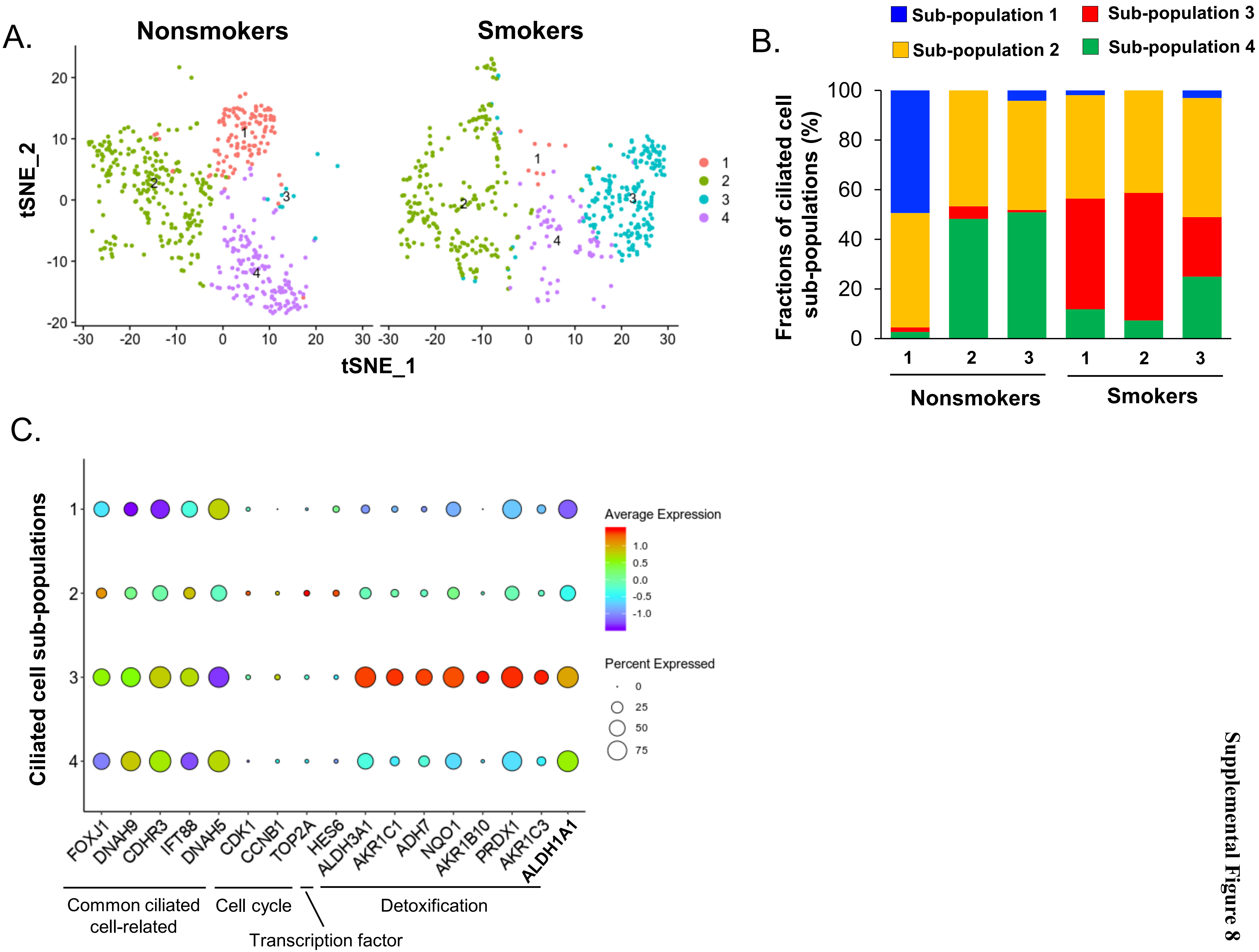


Supplemental Figure 8

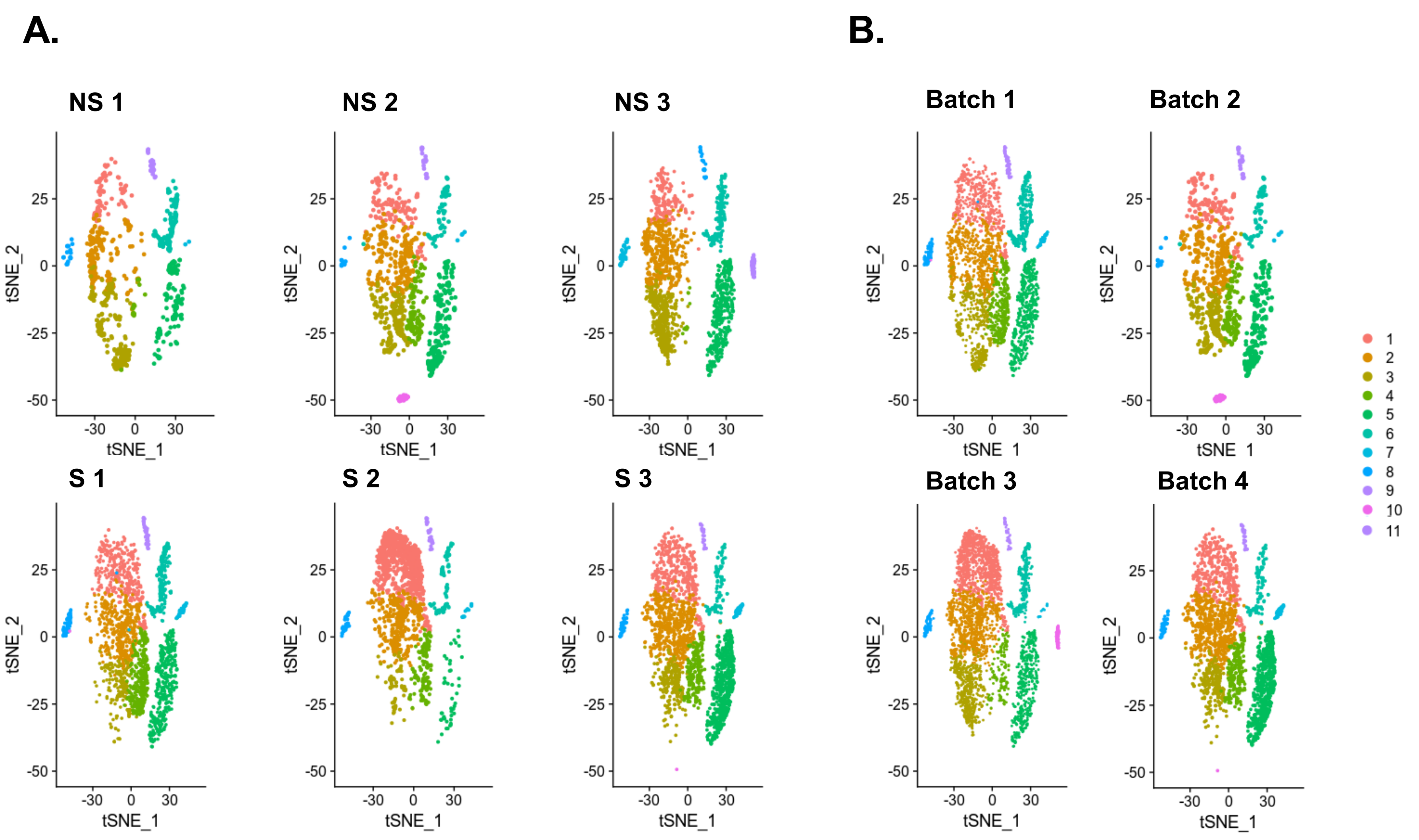


Supplemental Figure 9
